# Therapeutic potential of adenosine receptor modulators in cancer treatment

**DOI:** 10.1039/d5ra02235e

**Published:** 2025-06-17

**Authors:** Prasenjit Maity, Swastika Ganguly, Pran Kishore Deb

**Affiliations:** a Department of Pharmaceutical Sciences and Technology, Birla Institute of Technology Mesra Ranchi 835215 Jharkhand India prankishore1@gmail.com prankishoredeb@bitmesra.ac.in

## Abstract

All human cells contain the universal autocoid adenosine, which interacts with four types of G protein-coupled receptors (GPCRs), namely A_1_, A_2A_, A_2B_, and A_3_ adenosine receptors (ARs). Among these receptors, A_2A_ and A_2B_ ARs activate adenylate cyclase, while A_1_ and A_3_ ARs suppress the adenylate cyclase activity. Adenosine-receptor interactions play a crucial role in cancer biology by modulating the immune microenvironment, which tumors exploit to create immunosuppression that promotes their growth and metastasis. When the A_2A_ AR is activated on natural killer (NK) cells and T cells, it reduces their ability to carry out cytotoxic functions. This activation also encourages the formation of immune-suppressing cell types, such as myeloid-derived suppressor cells (MDSCs) and regulatory T cells (Tregs), further weakening the immune response. Targeting adenosine receptors, particularly the A_2A_ subtype, represents a promising therapeutic strategy. By antagonizing these receptors, it may be possible to restore T cell function, helping the body to recognize and attack cancer cells more effectively. Despite recent advancements in the discovery of novel, targeted anticancer agents, these treatments have shown limited effectiveness against metastatic tumours, complicating cancer management. Moreover, developing adenosine receptor agonists or antagonists with high target selectivity and potency remains a significant challenge, as the widespread distribution of adenosine receptors throughout the body raises concerns about off-target effects and reduced therapeutic efficacy. In order to improve outcomes for patients with advanced cancer, researchers are actively investigating safer and more efficient chemotherapy substitutes. However, drugs that activate A_3_ adenosine receptors and block A_2A_ receptors are being explored as a novel approach for cancer treatment. Monoclonal antibodies and small-molecule inhibitors targeting the CD39/CD73/A_2A_ AR axis are also being tested in clinical trials, both as standalone treatments and in combination with anti-PD-1/PD-L1 immunotherapies. This review primarily focuses on the signaling pathways and the therapeutic potential of various adenosine receptor agonists and antagonists across various cancer types, highlighting their ongoing evaluation in preclinical and clinical trials, both as monotherapies and in rational combination with immunotherapy, chemotherapy, or targeted therapies, potentially leading to the development of advanced treatments that could aid in tumor suppression.

## Abstract 2

Adenosine receptor signaling fosters an immunosuppressive environment that supports tumor growth and metastasis. Targeting these receptors alone or with other therapies holds promise for cancer treatment.

## Graphical abstract

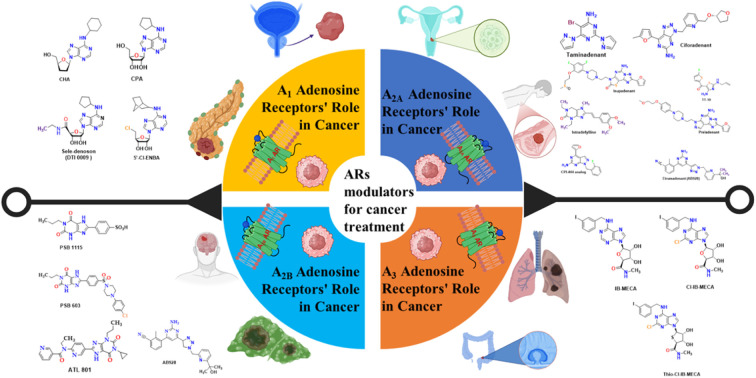

## 1. Introduction

**Fig. 1:**
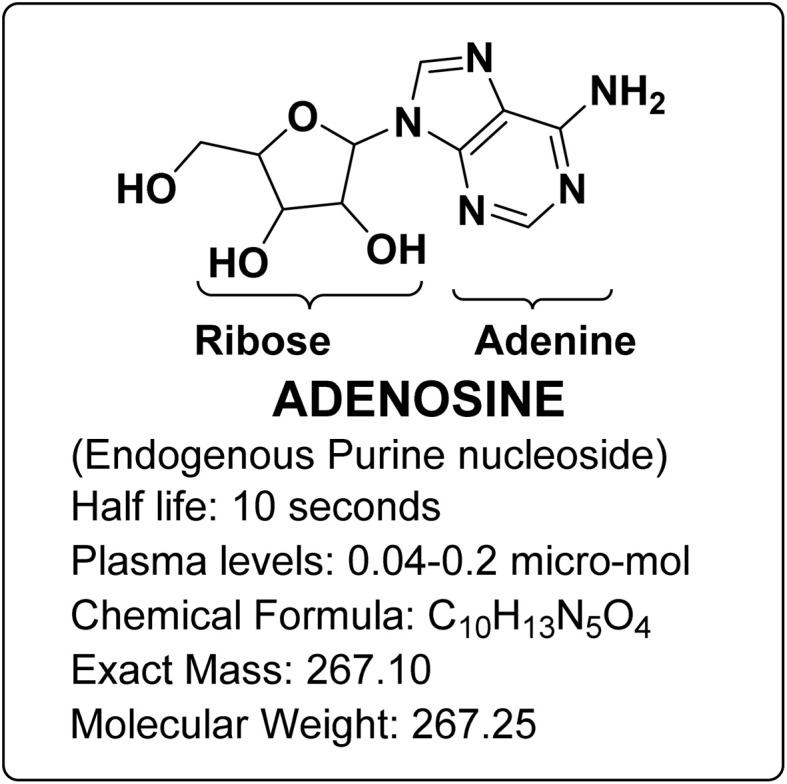
Structure of adenosine.

Adenosine is an endogenous purine nucleoside that comprises an adenine base linked to a sugar-containing ribose molecule *via* a β-N9-glycosidic bond, as depicted in Fig. 1.^1^ It serves as an essential element in human biology.^2^ Adenosine has been investigated for its potential role as a molecule with protective properties against cancer.^3^ Adenosine is well known for being an important local regulator of tissue function, particularly in situations where cellular energy demand exceeds the available energy supply.^4^ Adenosine is pivotal in maintaining cellular protection and modulating diverse physiological and pathological processes. In 1929, Drury and Szent-Gyorgyi elucidated adenosine's role as an extracellular signalling molecule, highlighting its broad impact on physiological functions.^5^

Adenosine exerts an important function in inhibiting the immune response against tumours by encouraging the growth of blood vessels (angiogenesis) and boosting the growth, progression, and mobility of tumour cells.^6,7^ However, there is ongoing debate about how adenosine specifically affects the death of cancer cells through apoptosis.^8^ As a result, ARs are considered promising targets for treating various medical conditions.^9–13^ Adenosine plays a role in the body's immune suppression in tumors. Some studies have shown that blocking adenosine or its receptors might lead to enhanced immunity against tumours, but it could also reduce the body's anti-tumour responses under certain conditions.^13^

Adenosine is a type of molecule derived from adenosine triphosphate (ATP) which is crucial for cellular energy. It serves as a significant regulator within the tumour microenvironment (TME). In conditions where there is low oxygen, limited blood flow, or inflammation, the level of adenosine increases in the region surrounding the tumour. This rise in adenosine can influence both the immune system's reactions and the tumor's growth dynamics.^14,15^

All four types of adenosine receptors have been identified as contributing to the progression of cancer.^10^ Numerous drug candidates that affect adenosine receptors in the body, including agonists, antagonists, partial agonists, and allosteric modulators, have been recently discovered and patented, and many of them are currently undergoing clinical trials.^14–23^

Recently, a growing focus has been on understanding the role of adenosine receptors' impact on cancer, affecting tumour growth, spread, and the immune system's response. Investigating these roles is vital for developing new treatments to combat cancer effectively. This review is focused on possible treatment strategies emphasizing the role of adenosine and its receptors in cancer development.

## 2. Adenosine & adenosine receptors

Adenosine is ubiquitous, released by nearly all cells, and produced in the extracellular environment through the breakdown of ATP by a cascade of ectoenzymes, including apyrase (CD39) and 5′-nucleotidase (CD73).^24^ When adenosine levels become excessive, the body has mechanisms to reduce them. Adenosine kinase can convert adenosine back into adenosine monophosphate (AMP) through phosphorylation, and adenosine deaminase (ADA) can deaminate adenosine into inosine.^16,17^ Both processes require sufficient oxygen to function effectively. However, these enzymes may not work efficiently in areas with low oxygen levels, such as in tumours affected by hypoxia. This can lead to the accumulation of adenosine in these regions, which can affect inflammation and contribute to tumour growth. Thus, the oxygen-dependent regulation of adenosine metabolism plays a crucial role in the tumour microenvironment.^17^

Elevated levels of CD73 have been noted in multiple cancer types, such as breast, colon, ovarian, melanoma, glioma, glioblastoma, leukemia, and bladder cancer.^18^

Adenosine, in turn, can act on immune cells and other cells in the tumour microenvironment, promoting immunosuppression and supporting tumour growth and metastasis. Therefore, CD73 expression in cancer cells is of interest as a potential target for therapeutic interventions to modulate the immune response against tumours.^19,25,26^

Adenosine is a potent compound that influences various cells and tissues, including platelets, coronary arteries, smooth muscle, cardiac muscle, and immune cells.^27^ As an extracellular messenger, it plays a role in conditions such as neurodegenerative diseases, psychiatric disorders, heart issues, lung injuries, cancers, and eye diseases.^28^

Adenosine receptors, encoded by separate genes, belong to the G protein-coupled receptor (GPCR) family. These receptors are categorized as A_1_, A_2A_, A_2B_, and A_3_ adenosine receptors (AR).^29,30^ The ARs are found throughout the human body in various organs and tissues, where they play crucial roles in regulating essential physiological functions, as shown in Table 1.^22,31–35^ Extracellular adenosine serves as a natural ligand for all these receptors. Each AR subtype exhibits unique binding affinities and is activated by adenosine in different ways, influencing diverse physiological processes.^36,37^ This interaction pattern allows adenosine to regulate neurotransmission, immune responses, inflammation, and vascular function across different tissues and organ systems in the body.^38^ The A_1_ AR, A_2A_ AR, and A_3_ ARs have moderate to high affinities for adenosine, and they usually require 1–10 nM, 30 nM and 100 nM concentrations of adenosine, respectively, for their activation. The A_2B_ AR, has the lowest affinity and requires a higher concentration of adenosine (approximately 1000 nM) for its activation.^10,23,39^

**Table 1:** The molecular features and functions of adenosine receptors in cells^a^

Receptors	Gene	Chromosomal location	Molecular weight (by amino acid sequence)/length (residues)	Affinity for adenosine (nM)	G-protein coupling	Signalling system	Effects on ion channels	Distribution		
								High expression	Intermediate expression	Low expression
A_1_ AR	ADO RA_1_	1q32.1	36 512/326	1–10	G_i_/G_o_	Block AC (↓cAMP), stimulate PLC (↑IP3/DAG), ↑PI3 kinase, ↑MAPK, ↑K^+^, Ca^2+^	↑K^+^ & ↓Ca^2+^	CNS, spinal cord, adrinal gland, atria	Adipose tissue, liver, renal tissue & skeletal muscle	In the higher bronchi & pancreas
A_2A_ AR	ADO R A_2A_	22q11.2	44 707/412	30	G_s_/G_olf_	Stimulate AC (↑cAMP), ↑MAPK	Inhibit Ca^2+^ channels	Lymphoid tissue (spleen, thymus, WBC & platelets)	Blood vessels, cardiac tissue, lung & peripheral nerve	Other regions of CNS
A_2B_ AR	ADOR A_2B_	17p11.2-12	36 333/332	1000	G_s_/G_q_	Stimulate AC (↑cAMP), activate PLC (↑IP3/DAG), ↑MAPK	Inhibit Ca^2+^ channels	Intraperitoneal pouch (cecum), urinary bladder, and colon	Lungs, eye, must cell & blood vessel	Adipose tissue, CNS, kidney & adrenal gland
A_3_ AR	ADOR A_3_	1p13.2	36 185/318	100	G_i_/G_q_	Block AC (↓cAMP), activate PLC (↑IP3/DAG), ↑PI3 kinase, ↑MAPK		Testis & must cell	CNS (hippocampus and cerebellum)	Liver, lymphatic tissue, thyroid and adrenal gland

a AC (adenyl cyclase), PLC (phospholipase C), G_i_/G_o_ (inhibitory G-proteins), DAG (diacylglycerol), IP3 (inositol triphosphate), G_s_/G_olf_ (stimulatory G-proteins), cAMP (cyclic adenosine monophosphate), K^+^ (potassium ion), Ca^2+^ (calcium ion), WBCs (white blood cells), CNS (central nervous system), PI3K (phosphoinositide 3-kinase), ADORA (adenosine receptor A), nM (nanomolar), MAPK (mitogen-activated protein kinase).

This similarity percentage gives us a rough estimate of how closely these receptors are related to each other; comparing the amino acid sequences of these receptors are roughly 49% of the residues in the A_1_ receptor are identical to those in the A_3_ receptor. Roughly 59% of the residues in the A_2A_ receptor are identical to those in the A_2B_ receptor.^37,40^

When adenosine binds to its receptors on the cell surface, it triggers a cascade of molecular events inside the cell. This includes activating MAPK (mitogen-activated protein kinase) proteins, which are enzymes that relay signals from the cell membrane to the nucleus. Once activated, MAPKs phosphorylate various target proteins involved in gene expression, cell cycle progression, and differentiation pathways.^41^ This pathway is essential for regulating fundamental cellular processes, including proliferation (cell division) and differentiation (maturation into specialized cell types). Various external signals can activate the MAPK pathway, with GPCRs playing a significant function in this process.^42^

AR, which is indeed a type of GPCR, regulates adenyl cyclase (AC) activity by either stimulating or inhibiting it. When AC is inhibited, it reduces the amount of cAMP in the cell. This affects the activity of PKA, which in turn phosphorylates proteins involved in the MAPK and AKT signalling pathways.^43,44^ The A_2A_ and A_2B_ receptors increase the activity of AC through the G_s_ protein. The A_2A_ receptor activates the G_s_ protein, while the A_2B_ receptor activates phospholipase C (PLC) through the G_q_ protein Which results in the formation of IP3 (Inositol trisphosphate) and DAG (Diacylglycerol).^45,46^

## 3. Purpose, mode of action, storage, release, and synthesis of adenosine

Adenosine is not stored in vesicles but is continuously released in response to metabolic changes. It modulates neuronal activity both presynaptically and postsynaptically. After acting, adenosine is either internalized for recycling or metabolized within the cell.^1,47^

**Fig. 2:**
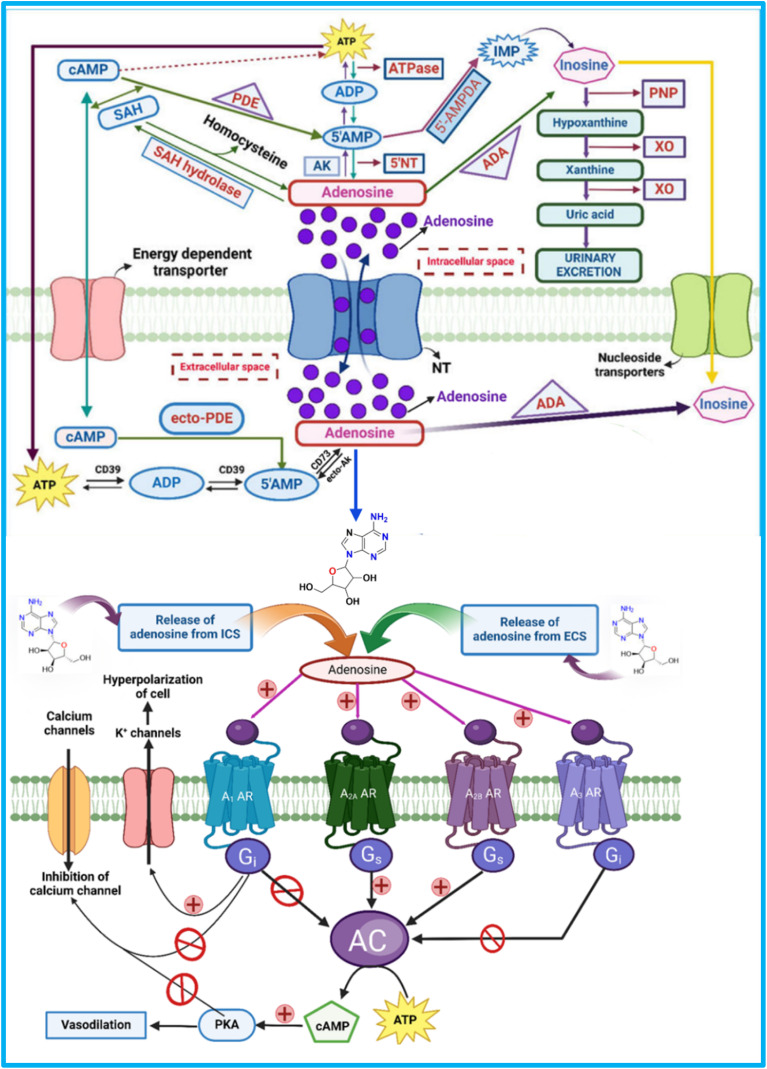
Synthesis, storage, release, and signalling pathways of adenosine through adenosine receptors. ATP – “Adenosine triphosphate”, CD73 – “Ecto-5′-nucleotidase” cAMP – “Cyclic adenosine monophosphate”, ADA – “Adenosine deaminase”, ADA – “Adenosine deaminase”, XO – “Xanthine oxidase”, CD39 – Ectonucleoside triphosphate diphosphohydrolase-1, PDE – “Phosphodiesterase”, SAH – “S-adenosyl-homocysteine”, 5-AMP – “5-Adenosine monophosphate”, PNP – “Purine nucleoside phosphorylase”, ADP – “Adenosine diphosphate”, 5NT – “5′-Nucleotidase”, ecto-AK – “Extracellular adenosine kinase”, A_1_ AR – “A_1_ adenosine receptor”, K^+^ channel – “Potassium channels”, A_2B_ AR – “A_2B_ adenosine receptor”, cAMP – “cyclic adenosine monophosphate”, G_i_ – “inhibitory G-proteins”, A_2A_ AR – “A_2A_ adenosine receptor”, AC – “Adenyl cyclase”, G_s_ – “Stimulatory G-proteins”, PKA – “Protein kinase A” A_3_ AR – “A_3_ adenosine receptor”. The figure was created in BioRender. Deb, P. (2025).

Adenosine is primarily produced extracellularly through the dephosphorylation of ATP, ADP, and AMP by two enzymes: CD39 (NTPDase 1), which converts ATP to ADP and AMP, and CD73 (5′-NT), which converts AMP to adenosine under stress (Fig. 2). Additionally, ecto-phosphodiesterase (ecto-PDE) enhances adenosine production by converting cAMP to AMP, further activating CD73.^48,49^

Once produced, adenosine is transported across the cell membrane by concentrative nucleoside transporters (CNTs) and equilibrative nucleoside transporters (ENTs).^50,51^ Equilibrative nucleoside transporters (ENTs) are membrane proteins that regulate intracellular nucleoside levels by facilitating their passive, bidirectional transport across the cell membrane *via* facilitated diffusion. They allow adenosine and other nucleosides to move in response to concentration gradients, helping maintain extracellular adenosine levels without ATP or ionic gradients.^52^

Inside the cell, adenosine undergoes key enzymatic transformations, including hydrolysis to form SAH by SAHH, phosphorylation to AMP by AK, and deamination to inosine by ADA. These processes are essential for regulating adenosine levels.^10,53,54^ The ecto-ADA removes extracellular adenosine and transports it inside cells *via* ENTs.^10,55–57^Fig. 2 represents the synthesis, storage, release, and signalling pathways of adenosine through adenosine receptors.

## 4. Adenosine's mode of action on its receptor subtypes

The affinity of each adenosine receptor for adenosine determines its activation, triggering G-proteins that regulate enzymes like adenylate cyclase (AC) to produce cAMP and modulate ion channels, affecting ion flow across the membrane. This regulation is crucial for intracellular signal transduction. Activating the A_1_ receptor by adenosine opens K^+^ channels, causing cell hyperpolarisation and inhibiting Ca^2+^ channels (Fig. 2), reducing calcium entry. This leads to vasodilation, lowering blood pressure, and increasing blood flow.^58^

Adenosine receptor activation affects cellular signalling by modifying critical pathways like cAMP production, activating PKA, and MAPK signalling, which influence gene expression, cell proliferation, and survival.^59–62^ The activation of the A_1_ AR inhibits AC and reduces cAMP levels. This diminishes PKA activity and CREB-1 phosphorylation, both crucial for gene regulation.^63,64^ A_1_ AR also activates MAPKs, including JNK, ERK1/2, and p38, which influence tumor growth and gene expression.^65–69^

Activation of A_2A_ AR triggers G_s_ protein signalling, which increases cAMP production *via* AC activation. PKA is activated by elevated cAMP, which also affects phosphodiesterases (PDES), CREB, and signalling pathways that control cell survival and proliferation. PKA also phosphorylates DARPP-32, modulating various cellular processes.^70,71^

A_2B_ AR directly affects essential proteins like p38 MAPK, ERK1/2, and JNK through its involvement in the MAPK signalling cascade. A_2B_ receptor activation plays a significant role in cancer cell growth and tumour progression by modulating stress-activated protein kinases (SAPK) and other MAPK-related processes.^72,73^

Through G_i_ protein signalling, A_3_ AR activation decreases cAMP levels and reduces PKA activity. As a result, increased activation of GSK-3β leads to decreased β-catenin, cyclin D1, and c-MYC expression, which inhibit cell growth and proliferation. Additionally, A_3_ AR suppresses NF-κB, which is involved in immune responses and inflammation.^74^

## 5. Role of adenosine and adenosine receptors on cancer

In cancer, the TME often exhibits higher levels of adenosine production, primarily due to reduced oxygen availability (hypoxia). Hypoxia promotes the breakdown of ATP, resulting in increased amounts of adenosine within the TME.^75^ The secretion of adenosine during hypoxic conditions stimulates angiogenesis, which in turn promotes tumor growth. In solid tumors, hypoxia enhances the production of CD39 and CD73, enzymes that generate adenosine, supporting tumor survival.^76,77^ Hypoxia activates transcription factors like HIF-1α, which regulate these enzymes, maintaining adenosine production under low-oxygen conditions.^78^ Elevated adenosine levels promote tumor growth, immune suppression, and angiogenesis by binding to A_1_, A_2A_, A_2B_, and A_3_ receptors.^79^

Adenosine accumulation in tumor hypoxic regions impairs immune cells' ability to target and destroy cancer cells. While lymphokine-activated killer (LAK) cell therapy shows promise for cancers resistant to standard treatments, its effectiveness is limited in colon cancer due to the tumor's immune-suppressive environment.^80,81^ Colon adenocarcinoma cells release a substance, distinct from TGF-β or prostaglandins, that inhibits the activation of anti-CD3-activated killer cells, helping the tumor evade immune attacks.^82^ In hypoxic tumor regions, adenosine accumulates and interacts with specific receptors on cytotoxic T cells, reducing their adhesion to cancer cells and impairing their activation.^82–84^ This effect, mediated by the A_3_ AR, inhibits cytotoxic T-cell activation and function in mice,^85^ contributing to immunosuppression and reduced efficacy of immunotherapy. Additionally, adenosine interferes with integrin α4β7, further impairing T cell adhesion to tumor cells.^86^

While adenosine's impact on other angiogenic factors is debated, it is known to boost the synthesis of certain phospholipases and support endothelial cell migration, further facilitating tumor growth and spread.^87,88^ Adenosine's capacity to stimulate the creation of PLCs and increase endothelial cell motility contributes to its involvement in promoting angiogenesis.^89,90^

## 6. A_1_ adenosine receptors' role in cancer: molecular signaling pathways

**Fig. 3:**
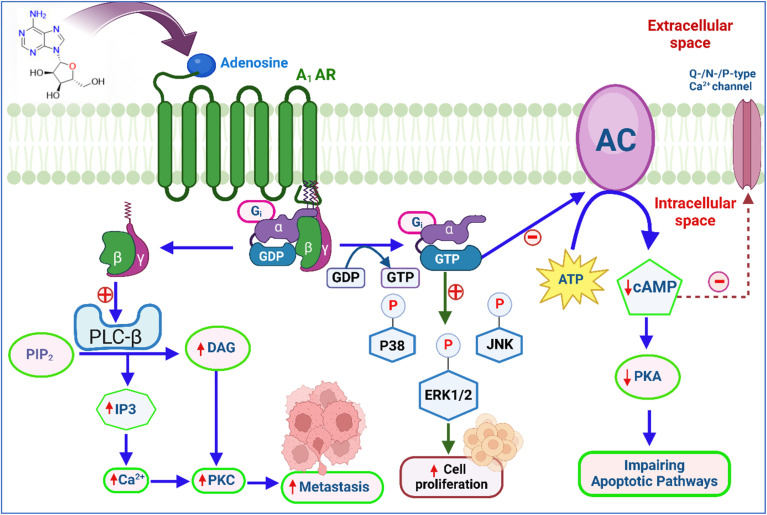
A_1_ adenosine receptors' role in cancer: molecular signaling pathways. GTP – “Guanosine triphosphate”, PKC – “Protein kinase C”, PKA – “Protein kinase A”, DAG – “Diacylglycerol”, GDP – “Guanosine diphosphate”, ERK1/2 – “Extracellular signal-regulated kinases”, ATP – “Adenosine triphosphate”, PIP2 – “Phosphatidylinositol 4,5-bisphosphate”, and IP3 – “Inositol trisphosphate”, cAMP – “Cyclic adenosine monophosphate” NK – “c-Jun N-terminal kinases”. The figure was created in BioRender. Deb, P. (2025).

The A_1_ adenosine receptor (A_1_ AR) plays several roles in cancer by influencing the TME. A_1_ AR signalling also affects cellular migration and invasion, potentially impacting metastasis and therapeutic resistance.^91^ Activation of the A_1_ AR stimulates phospholipase C-beta (PLC-β), which hydrolyzes PIP2 to produce IP3 and DAG. IP3 triggers calcium release from intracellular stores, activating calcium-dependent PKC and other calcium-binding proteins, impacting cellular functions.^10,64,92^ A_1_ AR activation also opens potassium channels, causing hyperpolarization and reducing cellular excitability, while inhibiting N-type and P/Q-type calcium channels (Fig. 3), further decreasing calcium influx and excitability in neurons and heart muscle.^93,94^

Overexpression of AC-3 increases molecules like matrix metalloproteinase 2 (MMP2), matrix metalloproteinase 9 (MMP9), and cAMP, promoting tumour growth.^68,69^ Inhibition of AC-3 slows tumor progression, while AC-2 is a marker for poor colon cancer outcomes. A_1_ receptor agonists reduce glioblastoma formation and microglial proliferation, and the absence of A_1_ AR increases microglial density around tumors.^95,96^

In brain tumors, hypoxic conditions within the TME cause adenosine levels to rise.^97^ This elevated adenosine activates theA_1_ AR in microglia, the brain's immune cells.^98^ Once A_1_ AR is activated, microglia become less effective at targeting and destroying cancer cells. This reduction in immune activity allows tumours to grow and spread more easily.^99^

The A_1_ AR and other adenosine receptors significantly aid tumor growth by promoting angiogenesis and forming new blood vessels. Tumors, especially in low-oxygen conditions, release high levels of adenosine which stimulates the production of vascular endothelial growth factor (VEGF) by interacting with A_1_ AR and other receptors on surrounding cells.^100^ VEGF then prompts endothelial cells to grow and form new blood vessels, which extend into the tumor, enhancing its blood supply, resulting in increased supply of nutrients and oxygen facilitating the tumor's growth and spread to other parts of the body.^100,101^

Blocking or modifying A_1_ AR function can counteract this suppression, enhancing the immune response and improving tumor control. This approach can be used alongside existing therapies, such as chemotherapy, radiation, or immunotherapy, to overcome resistance and improve patient outcomes. By addressing specific mechanisms of immune suppression, targeting A_1_ AR offers a promising strategy to enhance cancer treatment and survival rates.^102–104^

However, adenosine can also promote microglial proliferation through the combined action of A_1_ and A_2_ARs. While A_1_ ARs are often linked with suppressing immune responses, their interaction with A_2_ ARs can have different effects. Specifically, when adenosine activates both A_1_ and A_2_ ARs, it can stimulate the growth of microglia, which may alter the TME in ways that facilitate tumor growth and progression.^103^ A separate study found that blocking A_1_ AR prevented cell death caused by adenosine, while activating A_1_ AR led to the death of human colorectal carcinoma (CW2) cells. These findings imply that A_1_ AR contributes to the tumor-inhibitory properties of adenosine, suggesting that activation of this receptor may cause cancer cells to undergo apoptosis.^105^ The role of A_1_ AR in cancer was confirmed through studies where A_1_ AR levels were reduced in breast glands^106^ and kidney cancers.^107^ This was achieved using RNA interference and the A_1_ AR blocker DPCPX. Reducing A_1_ AR not only triggered cell death (apoptosis) in breast cancer cells but also slowed down tumour growth. Additionally, it caused cancer cells to stop progressing through the cell cycle at the G_2_/M phase and reduce the number of cells in the S phase, which is crucial for cell division.^106,108^

## 7. A_2A_ adenosine receptors' role in cancer: molecular signaling pathways

**Fig. 4:**
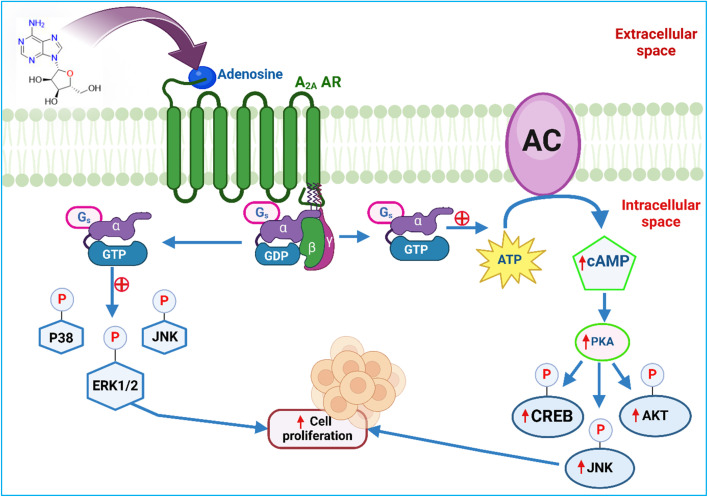
A_2A_ adenosine receptors' role in cancer: molecular signaling pathways. GTP – “Guanosine triphosphate”, CREB – “cAMP-response element binding protein”, JNK – “c-Jun N-terminal kinases”, ERK1/2 – “Extracellular signal-regulated kinases”, ATP – “Adenosine triphosphate” AKT – “Protein kinase B”, GDP – “Guanosine diphosphate”, and cAMP – “Cyclic adenosine monophosphate”. The figure was created in BioRender. Deb, P. (2025).

A_2A_ AR receptors play a key role in several cancer processes, including rapid cell proliferation, angiogenesis, immune escape, and metastasis.^109,110^ Thus, the increased presence of A_2A_ ARs enhances the cancer's ability to grow and metastasize uncontrollably (Fig. 4).^110^

Alternatively, the overexpression of A_2A_ ARs can also affect how immune cells, such as T cells, recognize and target cancer cells. Elevated levels of A_2A_ ARs can impair T cells' ability to effectively identify and attack tumour cells, as adenosine signalling through these receptors inhibits T cell activation and functionality,^111–113^ leading to suppression of immune cells, which in turn causes increased hypoxia within the tumour cells.^114^ Under hypoxic conditions, adenosine levels increase in the tumor microenvironment (TME), leading to immune suppression. When adenosine accumulates in the TME, it binds to the A_2A_ AR, and inhibits the immune system. This binding particularly impairs the function of tumor-reactive immune cells, such as T cells and natural killer (NK) cells^115,116^

## 8. A_2B_ adenosine receptors' role in cancer: molecular signalling pathways

**Fig. 5:**
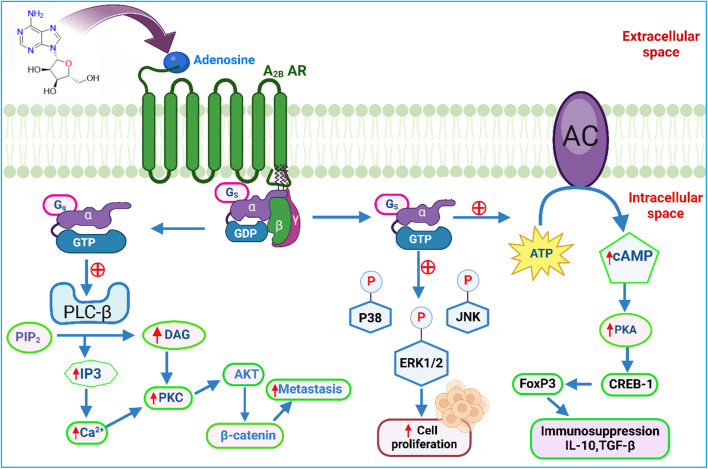
A_2B_ adenosine receptors' role in cancer: molecular signalling pathways. ATP – “Adenosine triphosphate”, CREB – “cAMP-response element binding protein”, PKC – “Protein kinase C”, IP3 – “Inositol trisphosphate”, GDP – “Guanosine diphosphate”, PLC-β – “phospholipase C-β”, IL-10 – “Interleukin 10”, AKT – “Protein kinase B”, ERK1/2 – “Extracellular signal-regulated kinases”, cAMP – “Cyclic adenosine monophosphate”, FoxP3 – “Forkhead box P3”, DAG – “Diacylglycerol”, PKA-“Protein kinase A”, GTP – “Guanosine trihosphate”, JNK – “c-Jun N-terminal kinases”, PIP2 – “Phosphatidylinositol 4,5-bisphosphate” and TGF-β “Transforming growth factor β”. The figure was created in BioRender. Deb, P. (2025).

Chinese hamster ovary (CHO) cells that produce recombinant human A_2B_ AR have been the primary and original demonstration source for classical A_2B_ AR signalling.^117–119^ Like the A_2A_ receptor, the A_2B_ AR is coupled with the G_s_ protein, which is crucial for intracellular signalling.^120^ Adenosine activates PKA (Fig. 5) by binding to A_2B_ AR, which phosphorylates target proteins and also recruits effectors like Epac (exchange protein directly activated by cAMP).^120–122^ This Epac impacts the proliferation of umbilical vein endothelial cells and triggers the expression of early genes, ultimately reducing smooth muscle cell growth in human coronary arteries.^123–125^ Additionally, A_2B_ AR is coupled with G_q_ proteins, which activate PLC that converts a molecule called PI2 into two important products: DAG and IP3. IP3 releases calcium ions within the cell, while DAG activates PKC.^126,127^ According to recent studies, activating A_2B_ AR reduces the activity of signalling molecules that are usually activated by the RANKL protein, such as p38, NF-κB (nuclear factor kappa-light-chain-enhancer of activated B cells), ERK1/2 (extracellular signal-regulated kinases 1 and 2), and p38. These signalling pathways collectively support tumour development and growth.^128^ Many studies have highlighted the importance of A_2B_ AR signalling in various disorders, including atherosclerosis,^129,130^ neurological inflammation,^131,132^ inflammatory bowel disease,^133^ ischemic heart preconditioning^134^ and the prevention of cardiovascular fibrosis. Overexpression of A_2B_ receptors is also linked to renal disorders.^135^

Wei *et al.* discovered that the A_2B_ AR is the most highly expressed adenosine receptor in human prostate cancer cell lines. According to their findings, TNFα and chemotherapy-induced cell death are lessened when the A_2B_ receptor is activated.^136^ J. Linden and colleagues showed the selective antagonist ATL801 to block the A_2B_ receptor, which prevents the development of 4T1 mammary carcinoma and MB49 urinary bladder tumours *in vivo*.^137^

## 9. A_3_ adenosine receptors' role in cancer: molecular signalling pathways

**Fig. 6:**
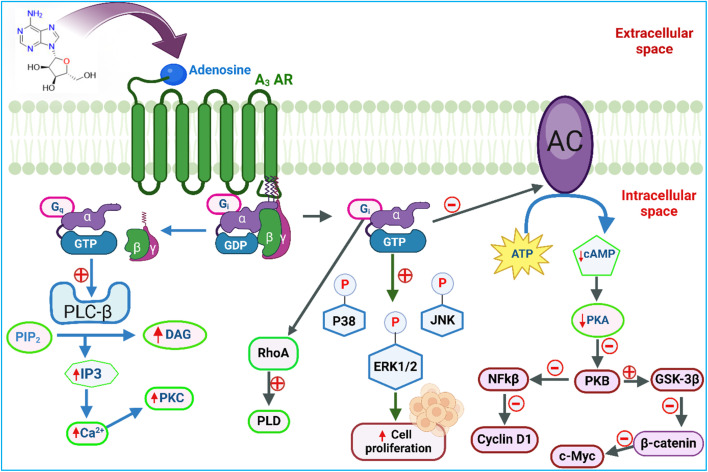
A_3_ adenosine receptors' role in cancer: molecular signaling pathways. ERK1/2 – “Extracellular signal-regulated kinases”, ATP – “Adenosine triphosphate”, PKA – “protein kinase A”, PIP2 – “Phosphatidylinositol 4,5-bisphosphate”, FoxP3 – “Forkhead box P3”, GSK-3β – “Glycogen synthase kinase-3β”, IP3 – “Inositol trisphosphate”, cAMP – “Cyclic adenosine monophosphate”, JNK – “c-Jun N-terminal kinases”, DAG “Diacylglycerol”, PKC – “Protein kinase C”, PKB – “Protein kinase B”, RhoA – “Ras homolog family member A” and NFκB – “Nuclear factor kappa-light-chain-enhancer of activated B cells”. The figure was created in BioRender. Deb, P. (2025).

The A_3_ AR is linked to G_q_ proteins that activate phospholipase C-β (PLC-β), producing IP3, which further increases intracellular calcium and activates PKC (Fig. 6).^10,138,139^

In normal tissue, A_3_ AR expression is minimal, but in tumour cells, it is markedly elevated;^140^ as a result, it can be regarded as a possible tumour marker. A_3_ AR levels are elevated in colorectal cancer and other malignant tumours, including pancreatic carcinoma, small-cell lung cancer, breast cancer, and melanoma, and are associated with disease progression.^141–144^ According to reports, human melanoma cells that express A_3_ AR signalling exhibit pro-survival effects.^145^ Glycogen synthase kinase (GSK)-3β, an enzyme essential for β-catenin phosphorylation, regulates this pathway, increasing tumour cell development.^146^ The expression of genes crucial for advancing the cell cycle, such as cyclin D1 and c-myc, is encouraged by phosphorylated β-catenin.^147^

Chromosome 1 at location p13.3 in humans contains the gene that codes for the A_3_ AR.^148^ A_3_ AR's overexpression in various tumour cells and tissues suggests that it may be useful as a therapeutic target and a cancer marker.^38,143^

## 10. Potential use of adenosine receptor ligands in cancer treatment

Selective modulators of adenosine receptors provide promising strategies for cancer treatment by directly inhibiting tumour growth or modifying the tumour microenvironment to enhance anti-tumour immune responses. These modulators target specific adenosine receptors: A_1_, A_2A_, A_2B_, and A_3_, each playing distinct roles in cancer progression (Table 2). Their effects and mechanisms in cancer treatment are actively being explored, and future research will likely continue to refine these pathways for more targeted and effective therapies. Below is an elaboration on the selective modulators for each receptor type, their effects, and mechanisms in cancer treatment.

**Table 2:** Some selective modulators for various types of adenosine receptors, along with their effects and mechanisms of action in cancer treatment

S. No.	Name of the compounds	Agonist/antagonist	Target receptor subtype	Transduction mechanism	Description role
1	CHA	Agonist	A_1_ receptor	AC ↓ PLC ↑	Binds to A_1_AR and activates it; this activation leads to decreased AC and decreased cAMP in the cell
2	CPA	Agonist	A_1_ receptor	AC ↓ PLC ↑	Activated G_i_ proteins inhibit AC activity, decreasing the production of cAMP from ATP. Lower cAMP levels result in reduced activation of PKA and downstream signaling pathways
3	CCPA	Agonist	A_1_ receptor	AC ↓ PLC ↑	cAMP levels can lead to decreased activation of protein kinase A (PKA), which promotes cell growth and proliferation
4	S-ENBA	Agonist	A_1_ receptor	AC ↓ PLC ↑	cAMP levels due to A_1_AR activation can lead to decreased activation of protein kinase A (PKA)
5	5′-Cl-ENBA	Agonist	A_1_ receptor	AC ↓ PLC ↑	Can reduce PKA activity, potentially inhibiting the proliferation of cancer cells
6	Sele-denoson or DTI 0009	Agonist	A_1_ receptor	AC ↓ PLC ↑	Lowering AC level
7	GR79236X	Agonist	A_1_ receptor	AC ↓ PLC ↑	Decrease the cAMP level
8	Tiazofurin	Agonist	A_1_ receptor	AC ↓ PLC ↑	Inhibit inosine monophosphate dehydrogenase (IMP dehydrogenase) activity so, it can decrease the levels of downstream purine nucleotides, which can impact cell proliferation, especially in rapidly dividing cells such as cancer cells
9	Cyclosaligenyl-tiazofurin monophosphate	Agonist	A_1_ receptor	AC ↓ PLC ↑	Reduction in cAMP levels may affect downstream effectors, such as protein kinase A (PKA), which can influence cell growth
10	1,3-Dipropyl-8-cyclopentylxanthine	Antagonist	A_1_ receptor	AC ↑	The inhibitory effect that adenosine has on adenylate cyclase, increased adenylate cyclase activity and higher levels of cAMP within the cell
11	HENECA	Agonist	A_2A_ receptor	AC ↑	Activating A_2A_ receptors may influence the immune response within the tumor microenvironment
12	CGS15943	Antagonist	A_2A_ receptor	AC ↓	Binding to the A_1_ receptor usually results in reduced cyclic AMP (cAMP) levels due to inhibition of adenylate cyclase
13	ZM241385	Antagonist	A_2A_ receptor	AC ↓	Antagonising A_2A_ adenosine receptors, leading to decreased cAMP production
14	SCH58261	Antagonist	A_2A_ receptor	AC ↓	Promote T cell function and enhance anti-tumour immune responses
15	SCH-442416	Antagonist	A_2A_ receptor	AC ↓	Blocking the A_2A_ receptor
16	SYN115 (tozadenant)	Antagonist	A_2A_ receptor	AC ↓	Inhibition of the A_2A_ receptor can disrupt pathways that allow cancer cells to evade immune surveillance, potentially leading to reduced tumor growth
17	TP455	Antagonist	A_2A_ receptor	AC ↓	Inhibition of A_2A_ receptors can lead to increased activation and proliferation of T cells
18	CPI-444	Antagonist	A_2A_ receptor	AC ↓	Inhibits the A_2A_ adenosine receptor
19	PBF-509	Antagonist	A_2A_ receptor	AC ↓	The antagonism of A_2A_ receptors may also help inhibit pathways that contribute to tumor cell survival and proliferation
20	AZD4635	Antagonist	A_2A_ receptor	AC ↓	Blocking the A_2A_ receptor
21	TT-10	Antagonist	A_2A_ receptor	AC ↓	Inhibits the A_2A_ adenosine receptor
22	Preladenant	Antagonist	A_2A_ receptor	AC ↓	Inhibits the A_2A_ receptor
23	Etrumadenant (AB928)	Antagonist	A_2A_ receptor	AC ↓	Blocking A_2A_ receptors helps to counteract the immunosuppressive effects of adenosine
24	Inupadenant	Antagonist	A_2A_ receptor	AC ↓	Inhibits the A_2A_ AR
25	ANR 94	Antagonist	A_2A_ receptor	AC ↓	Selectively inhibits the A_2A_ receptor
26	CPI-444 analog	Antagonist	A_2A_ receptor	AC ↓	Inhibition of A_2A_ receptors
27	Istradefylline	Antagonist	A_2A_ receptor	AC ↓	Inhibition of A_2A_ receptors
28	PSB1115	Antagonist	A_2B_ receptor	AC ↑	Inhibit A_2B_ receptor
29	PSB603	Antagonist	A_2B_ receptor	AC ↑	Blocked A_2B_ receptor
30	ATL801 (30)	Antagonist	A_2B_ receptor	AC ↑	A_2B_ receptor blocked
31	AB928	Antagonist	A_2B_ receptor	AC ↑	A_2B_ receptor inhibitor
32	Piclidenoson (IB-MECA)	Agonist	A_3_ receptor	AC ↓ PLC ↑	Boost the activity of regulatory T cells (Tregs), thereby increasing immunosuppression within the TME
33	Namodenoson (Cl-IB-MECA)	Agonist	A_3_ receptor	AC ↓ PLC ↑	Promoting anti-tumor immunity by reducing inflammation while also potentially inducing apoptosis in tumor cells
34	Thio-Cl-IB-MECA	Agonist	A_3_ receptor	AC ↓ PLC ↑	Thio-Cl-IB-MECA binds to the A_3_ adenosine receptor. Upon activation, the A_3_ receptor inhibits adenylate cyclase, leading to decreased levels of cAMP in the cell
35	Cordycepin	Agonist	A_3_ receptor	AC ↓ PLC ↑	A_3_ receptor activation
36	*N* ^6^-(2-Isopentenyl) adenosine	Agonist	A_3_ receptor	AC ↓ PLC ↑	Activation of A_3_ receptors lead to the inhibition of cell proliferation
37	Resveratrol-3-*O*-d-glucuronide	Agonist	A_3_ receptor	AC ↓ PLC ↑	A_3_ receptor activation
38	Resveratrol 4′-*O*-d-glucuronide	Agonist	A_3_ receptor	AC ↓ PLC ↑	A_3_ receptor activation
39	Linagliptin	Agonist	A_3_ receptor	AC ↓ PLC ↑	A_3_ receptor activation
40	Oxidative degradation product of linagliptin	Agonist	A_3_ receptor	AC ↓ PLC ↑	A_3_ receptor activation
41	MRS 1523	Antagonist	A_3_ receptor	AC ↑	Inhibit A_3_ receptor
42	Truncated thio-Cl-IB-MECA	Antagonist	A_3_ receptor	AC ↑	Inhibit A_3_ receptor
43	*N* ^6^-(2,2-Diphenylethyl)-2-phenylethynylAdo	Antagonist	A_3_ receptor	AC ↑	Inhibit A_3_ receptor
44	[1,2,4]-Triazolo[1,5-*c*]pyrimidines	Antagonist	A_3_ receptor	AC ↑	Inhibit A_3_ receptor
45	MRS 1097	Antagonist	A_3_ receptor	AC ↑	Inhibit A_3_ receptor
46	MRS 1067	Antagonist	A_3_ receptor	AC ↑	Inhibit A_3_ receptor
47	MRS 1220	Antagonist	A_3_ receptor	AC ↑	Inhibit A_3_ receptor
48	MRS 1191	Antagonist	A_3_ receptor	AC ↑	Inhibit A_3_ receptor

**Fig. 7:**
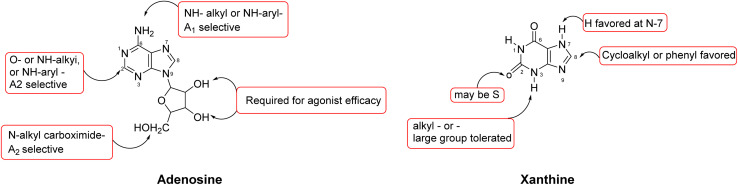
Impact of structural modifications of adenosine and xanthine derivatives on adenosine receptor binding.

Many studies have been conducted on the structure–activity relationship (SAR) of adenosine derivatives as agonists of the AR. Adenosine and xanthosine, two purine nucleoside derivatives, constitute the basis for the majority of known AR agonists. Many agonists that target distinct AR subtypes have been developed due to modifications to the physiological agonist adenosine. Adenosine's affinity for adenosine receptors is increased by adding nitrogen atoms at positions 3 and 7.^149^ Mono-substitution of the *N*^6^ position (exocyclic amino group) of adenosine with a large, non-polar (hydrophobic) group enhances the molecule's ability to preferentially bind to the A_1_ and A_3_ AR subtypes.^33,149^ The existence of a hydrogen atom at position *N*^6^ is critical for agonist activation because it creates hydrogen bonds with the receptor.^150^ For tiny halogen atoms or bigger substituents, modifications to adenine at the *C*_2_ position are generally well tolerated.^151^ Strong and focused agonistic action at ARs is produced by substitutions at the 5′-position, which are often well tolerated and require the presence of a ribose sugar moiety. In the adenosine structure, the 2′- and 3′-hydroxyl (OH) groups of the ribose moiety are essential for agonist activity at adenosine receptors (Fig. 7).

Structure–activity relationship (SAR) studies at adenosine receptors show that xanthines with strong inhibitory activity were consistently alkylated at the 1-position, where substituting hydrogen with a methyl group enhanced potency by approximately 20-fold. Modifications at the 3-position were not essential for high activity. Substitution at the 7- and 8-position might slightly increase or decrease the potency, while substitutions at the 7-position are typically considered unfavourable. However, certain alkyl modifications at these positions can selectively boost affinity for the A_2_ receptor subtype.^152^

### 10.1 The A_1_ receptor agonists

The selectivity towards A_1_ AR was found to be increased mainly upon the introduction of a larger cycloalkyl group at the *N*^6^-position of the agonists like CHA (*N*^6^-cyclohexyladenosine) (1) and CPA (*N*^6^-cyclopentyladenosine) (2), being 400- and 800-fold selective.^153^ Combined substitutions at the *N*^6^- and 2-positions have yielded 2-chloro-CPA (CCPA) (3), which is 1500-fold A_1_-selective, and helpful in wide pharmaceutical applications.^33,149–151,154,155^

Mice deficient in A_1_ AR and their wild-type littermates responded differently to injections of Gl261 glioblastoma tumour cells and treatment with adenosine and *N*^6^-cyclopentyladenosine (CPA), where CPA significantly reduced the growth of the tumour. Remarkably, research revealed that in brain slices taken from A_1_ AR-deficient mice, neither adenosine nor CPA (*N*^6^-cyclopentyladenosine) had any impact on tumour formation. These results suggest that CPA and adenosine influence tumour size reduction by selectively interacting with A_1_ ARs on microglial cells.^96^

Like numerous other *N*^6^-substituted adenosine analogues, both CPA and CCPA derivatives show notable affinity for the A_3_ AR as well. In contrast to A_1_ AR, CCPA has been identified as an antagonist at the human A_3_ AR, with a *K*_i_ value of 35 nM.^156^*N*^6^-Bicycloalkyladenosine is even more A_1_-selective, with S-ENBA (4), showing 4700-fold selectivity for the A_1_ receptor.^157–159^ Among the most selective A_1_ AR agonists, 5′-Cl-ENBA (5) demonstrates exceptionally high affinity and selectivity for the A_1_ AR compared to other AR subtypes.^160,161^ Through the activation of A_1_ AR, *N*^6^-cyclopentyl-NECA, often referred to as sele-denoson or DTI 0009 (6), exhibited the strongest negative dromotropic impact (A_1_ AR).^162^ Seledenoson has been tested in phase 2 clinical trials to determine its efficacy in treating atrial fibrillation *via* oral and intravenous (IV) routes.^163^ In addition the other A_1_-selective adenosine derivatives, including GR79236, clinical investigations have examined the possible use of *N*^6^-[(1*S*, *trans*)-2-hydroxy-cyclopentyl] adenosine (GR79236X) (7), a hydroxylated derivative of CPA, in the treatment of myocardial ischemia, diabetes, and pain.^33^

**Fig. 8:**
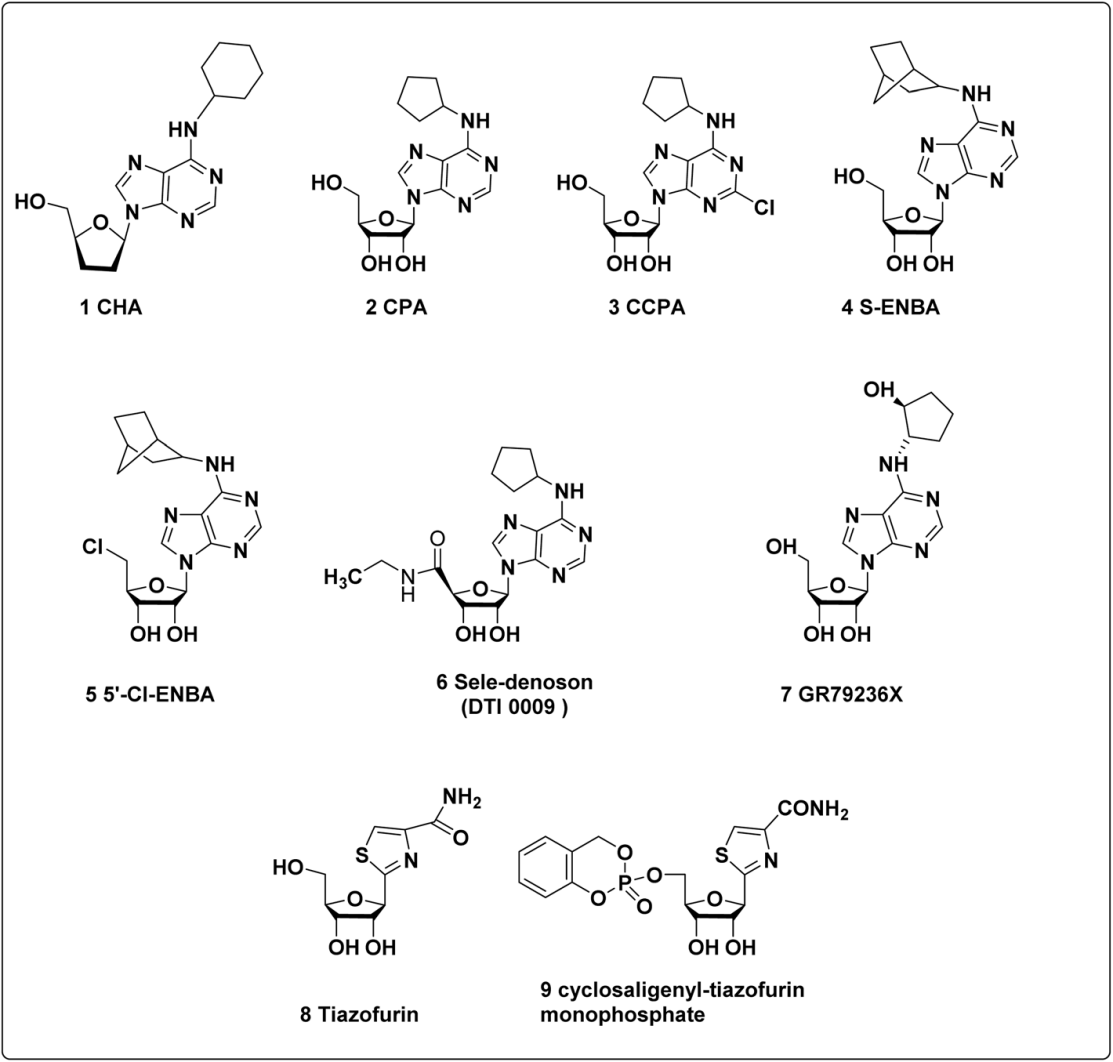
Potential A_1_ adenosine receptor agonists exhibiting promising anticancer properties.

Tiazofurin (8) is a C-nucleoside that inhibits inosine monophosphate dehydrogenase (IMPDH) and has demonstrated anticancer efficacy in clinical settings. Tiazofurin adenine dinucleotide, produced when metabolized, inhibits the IMPDH enzyme, stopping nucleotide synthesis and inhibiting the growth of cancerous cells.^108^ A new tiazofurin pronucleotide called cyclosaligenyl-tiazofurin monophosphate (9) is a selective agonist of the A_1_ AR. Its binding affinity is comparable to that of tiazofurin, suggesting a similar mechanism of action at this receptor subtype. Additionally, it exhibits significant efficacy against the K-562 cell line, which is a model for human chronic myelogenous leukaemia (*in vitro*). Structural representations of interesting A_1_ AR agonists with anti-cancer activity are shown in Fig. 8.

### 10.2 The A_1_ receptor antagonists

**Fig. 9:**
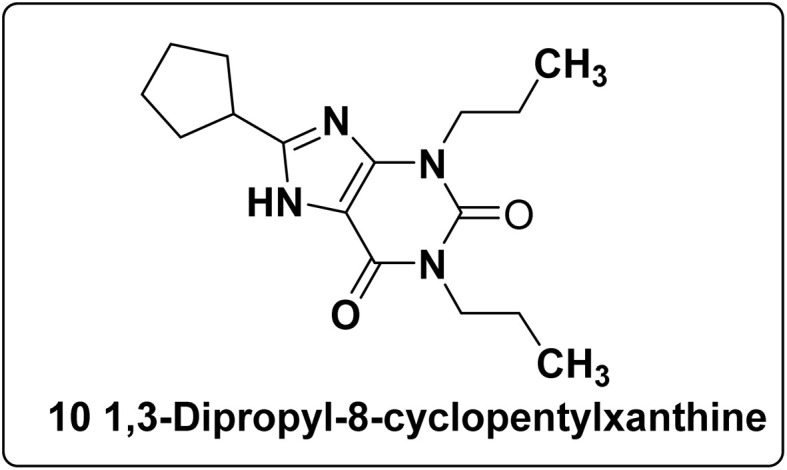
Potential A_1_ adenosine receptor antagonist exhibiting promising anticancer properties.

The A_1_ AR antagonist has diverse effects on different forms of cancer and may help in preventing the development of specific cancer types. Recent investigations have employed quantitative real-time PCR and western blotting analysis to ascertain the function of A_1_ AR in kidney cancer in 786-O & ACHN cell lines. In addition, investigations on anticancer research have shown that 1,3-dipropyl-8-cyclopentylxanthine (10), an A_1_ AR antagonist (Fig. 9), efficiently decreases the formation of tumours *in vivo* and the proliferation of RCC cells *in vitro*. Compound 10 also inhibited the migration of RCC cells; however, the selective A_1_ agonist *N*^6^-cyclopentyladenosine (CPA) increased the migration of RCC cells. Furthermore, the xanthine derivative 10 produced an arrest in the S-phase of the cell cycle and increased apoptosis in 786-O and ACHN cells.^108^

### 10.3 The A_2A_ receptor selective agonists

**Fig. 10:**
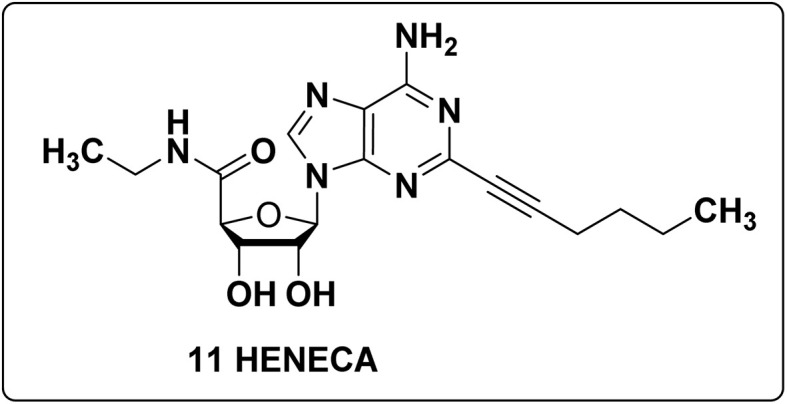
Potential A_2A_ adenosine receptor agonist exhibiting promising anticancer properties.

With A-375 cells, the anticancer effects were studied using a particular A_2A_ agonist, (2*S*,3*R*,5*R*)-HENECA (2-hexynyl-NECA) (11) as shown in Fig. 10, which demonstrated consistent, modest effects in inhibiting cell proliferation and lowering cytotoxicity in A-375 cell lines.^106,164^ Compound 11 causes concentration-dependent cell death, with peak effects being shown at 100 nM. Higher concentrations, however, resulted in a decrease in its effectiveness.

### 10.4 The A_2A_ receptor-selective antagonists

**Fig. 11:**
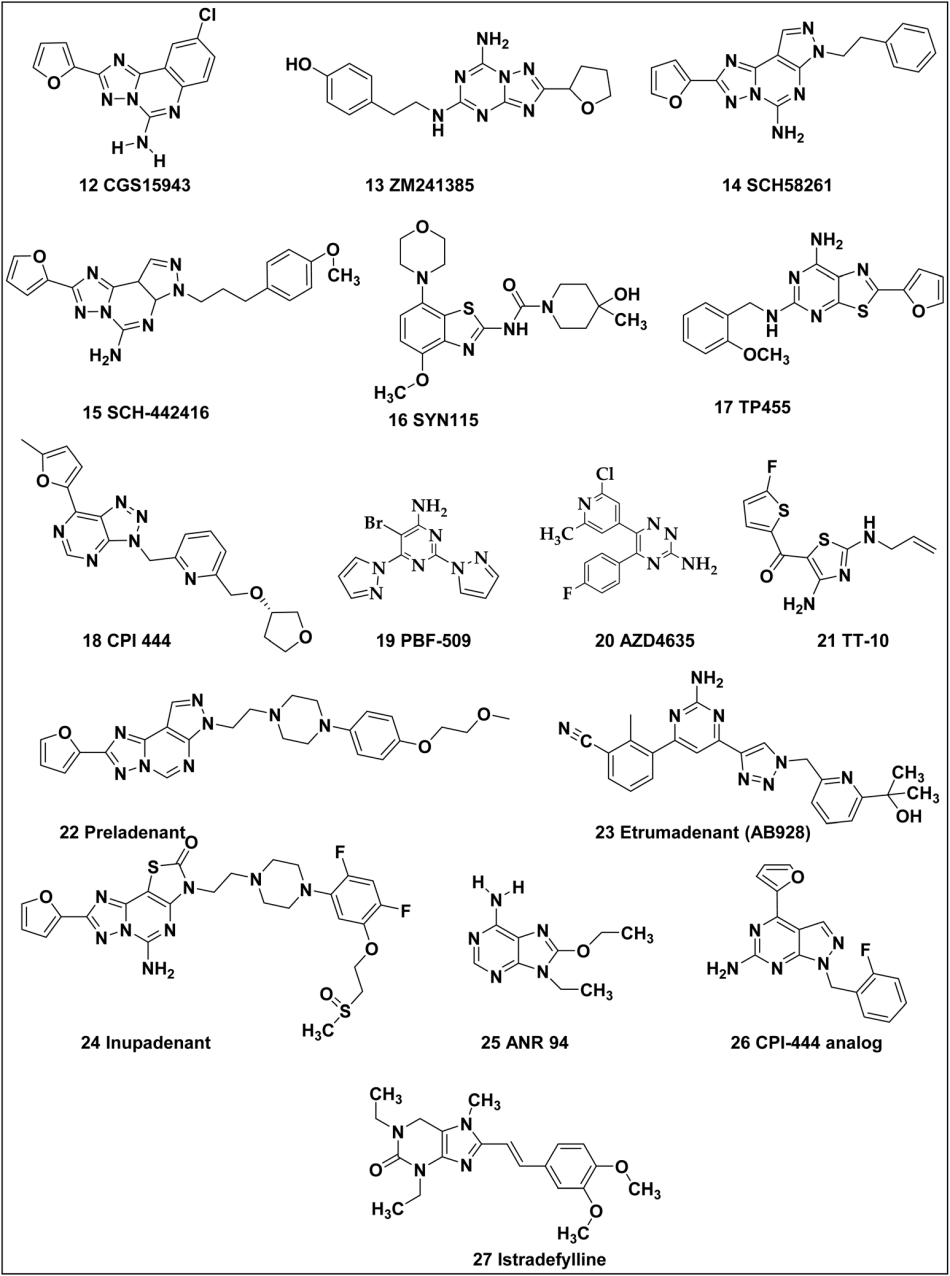
Potential A_2A_ adenosine receptor antagonists exhibiting promising anticancer properties.

Hypoxic solid tumours are known to include an increased amount of adenosine, which hinders the capacity of cytolytic T lymphocytes that are essential to identify cancer cells as targets.^112,165^ It has been discovered that the activation of A_2A_ AR stimulates angiogenesis and boosts the growth of melanoma and mammary cells. These findings emphasise the significance of developing A_2A_ AR antagonists for the treatment of cancer.^166,167^ Examples of A_2A_ AR antagonists with anticancer effects are shown in Fig. 11.

The effects of adding styryl groups to xanthines at the 8-position have been brought to light by recent developments in search of A_2A_ AR antagonists. This has increased the A_2A_ AR selectivity.^168–170^

However, replacing the xanthine core with various heterocyclic ring systems has resulted in exceptionally high affinity and selectivity for the A_2A_ AR. An early example of a heterocyclic structure proposed as an A_2A_ AR antagonist was triazoloquinazoline (CGS15943) (12), which was later found to exhibit only modest selectivity. Subsequent modifications, such as adding a third ring or altering the nitrogen arrangement within the heterocyclic system, significantly enhanced A_2A_ AR selectivity.^5,171^ Triazoloquinazoline CGS15943 (12), an early example of a heterocyclic antagonist, was discovered to have only moderate selectivity, contrary to initial predictions.

Selectivity for the A_2A_ AR was markedly increased by further modifications to the triazoloquinazoline, with adjustments to the nitrogen arrangement within the heterocyclic structure leading to the development of a compound with improved potency, triazolotriazine ZM241385 (13).^172^

ZM241385 “(4-(2-((7-amino-2-(furan-2-yl)-[1,2,4]triazolo[1,5-*a*][1,3,5]triazin-5-yl)amino)ethyl)phenol)” is an A_2A_ AR antagonist that enhances the ability of antitumor T lymphocytes to suppress tumour growth, eradicate cancer, and prevent the production of new blood vessels. Compound 13 stimulated the development of anti-CL8-1 CD8^+^ T cells and markedly slowed the growth of CL8-1 tumours in wild-type mice. When this antagonist (compound 13) is used in conjunction with anti-CD8^+^ T cells in adoptive immunotherapy models, tumour growth is successfully prevented; however, the antagonist by itself does not have the same impact.^170^ In melanoma-bearing mice, the efficacy of compound 13, was investigated in combination with an anti-CTLA4 monoclonal antibody.^173^ Tumor growth was significantly inhibited in mice receiving just compound 13 treatment. In contrast to either the control group or treatment with compound 13 alone, the combination therapy led to a more noticeable delay in the progression of the tumor. There is a theory that this enhanced effect is linked to a decrease in regulatory T cells (TREGs) and an increase in CD8-positive (CD8^+^) T lymphocyte infiltration within the tumor tissue. In cutaneous melanoma tissue, treatment with the compound 13 alone resulted in increased infiltration of CD8^+^ T cells and a decrease in regulatory T cells (TREGs).^168^

Interestingly, the pyrazolotriazolopyrimidine group plays a significant role in improving A_2A_ AR selectivity. In SCH58261 (14), the xanthine core is also modified, but it includes additional aromatic and heteroatom-containing groups. SCH58261 (14) “2-(furan-2-yl)-7-phenethyl-7*H*-pyrazolo[4,3-*e*][1,2,4]triazolo[1,5-*c*]pyrimidin-5-amine” is an A_2A_ receptor blocker that has been studied for its potential to prevent metastasis using the 4T1.2 cancer model, a highly aggressive metastatic mammary gland tumor cell line.^174^ Research has demonstrated that by blocking the A_2A_ AR, the triazolo-pyrimidine derivative 14 dramatically reduces cancer cell proliferation in B16F10 CD73^+^ malignancies. Additionally, compared to the control group, therapy with the A_2A_ antagonist 14 resulted in a significant reduction in the metastasis of 4T1.2 tumors. Moreover, A_2A_−/– animals showed significant defence against B16-F10 CD73^+^ tumor cell metastasis.^175^ In non-A_2A_−/– animals, the A_2A_ antagonist 14 likewise showed decreased efficacy, demonstrating the drug's specificity for the A_2A_ receptor.^175,176^ In animal models of skin and breast tumor, compound 14 was also demonstrated to improve survival and lessen the stress caused by metastatic cancer when given in combination with an anti-PD-1 monoclonal antibody.^176^

Incorporating pyrazole and triazole rings into the structure of SCH442416 (15) enhances its binding affinity by altering the electronic properties of the xanthine core. These modifications improve interactions with the A_2A_ receptor.^29^ Specifically, the addition of an N′-substituted pyrazolotriazolopyrimidine group refines the molecule's conformation, increasing A_2A_ receptor selectivity while reducing affinity for other adenosine receptor subtypes. As a result, SCH442416 exhibits high affinity for the A_2A_ receptor (hA_2A_*K*_i_ = 1.1 nM) and significantly lower affinity for the A_1_ receptor (hA_1_*K*_i_ = 549 nM), establishing it as a widely used reference antagonist for A_2A_ receptor studies.^177^

In a follow-up investigation, researchers evaluated the benzothiazole derivative SYN115 (tozadenant) (16) for its potential to act as an antagonist of the A_2A_ AR. The study focused on how this property could enhance the anticancer effects of an anti-PD-1 monoclonal antibody, which is used in cancer immunotherapy. By blocking the A_2A_ receptor, SYN115 may help improve immunological reaction to malignancies, thereby possibly increasing the effectiveness of the anti-PD-1 treatment in combating cancer. It was demonstrated that the A_2A_ receptor blocker 16 significantly increased the anticancer efficacy of the anti-PD-1 antibody, with results comparable to compound 15.^70^

The role of A_2A_ AR in a number of human cancers, such as rat MRMT-1 breast cancer, A-375 melanoma, and A-549 lung cancer, has been extensively studied. Special attention has been paid to the signaling pathways involved and the outcomes of a novel A_2A_ receptor antagonist, TP455 (17) (2-(furan-2-yl)-*N*5-(2-methoxybenzyl)thiazolo[5,4-*d*]pyrimidine-5,7-diamine), as well as the actions of this antagonist.^178^

The immune-activating and anti-tumor properties of (*S*)-7-(5-methylfuran-2-yl)-3-((6-(((tetrahydrofuran-3-yl)oxy)methyl)pyridin-2-yl)methyl)-3*H*-[1,2,3]triazolo[4,5-*d*]pyrimidin-5-amine, CPI-444 (18), either alone or in combination with anti-PD-1/PD-L1 monoclonal antibodies, have been evaluated *in vitro* using activated primary human T cells. The results showed that pyrimidine derivative 18 completely inhibited the production of intracellular cAMP when the cells were incubated with 5′-*N*-ethylcarboxamidoadenosine (NECA), a stable adenosine analog. Activated T lymphocytes produce less IL-2 and IFN-γ when the A_2A_ AR agonist prevents fast TCR-mediated ERK phosphorylation. On the other hand, T cell signalling and function were restored when antagonist 17 blocked the A_2A_ AR. Efficacy of the compound 18 has been examined using CT26 and MC38 syngeneic animal tumor models. The absence of fresh tumour development in cured animals when re-challenged with MC38 cells indicates that antagonist 18 produced systemic anti-cancer immunological memory. Molecule 17, in conjunction with an anti-PD-L1 monoclonal antibody in the MC38 model, led to a synergistic decrease in tumour development and total tumour eradication in 9 out of 10 treated mice. In the CT26 model, antagonist 17 and anti-PD-1 monoclonal antibody also showed synergistic effects; the combination dramatically reduced tumour development and increased survival over antagonist 17 treatment alone.^179,180^

The A_2A_ AR-mediated immunological checkpoint is activated when adenosine levels rise in the tumour microenvironment, suppressing anti-tumour responses. Enhancing anti-tumour T cell function may be possible by focusing on this immunological checkpoint. PBF-509 (5-bromo-2,6-di(1*H*-pyrazol-1-yl)pyrimidin-4-amine) (19) is a novel A_2A_ AR antagonist that has been developed as a possible treatment for non-small cell pulmonary carcinoma in this regard.^181^ According to research, PBF-509 has strong selectivity for the A_2A_ AR. PBF-509 therapy decreased lung metastases in a mouse model compared to the control group. Additionally, among recently extracted tumor-infiltrating lymphocytes from lung cancer patients, investigations showed varied expression of the A_2A_ receptor in CD8^+^ cells and enhanced expression in CD4^+^ cells. Additionally, when PBF-509 was administered in conjunction with anti-PD-1 or anti-PD-L1 therapy, human tumor-infiltrating lymphocytes showed enhanced reactivity, according to *in vitro* investigations.^182^ These results imply that blocking the A_2A_ AR may open up new avenues for the development of creative immunotherapeutic approaches to treat non-small-cell lung cancer.^183^

The use of A_2A_ AR antagonists in the treatment of cancer, either as monotherapy or in addition to different immunotherapies, is now being investigated in a number of clinical trials, which is relevant.^91,184^ A number of A_2A_ AR antagonists are currently being evaluated in clinical settings including AZD4635 (20). In TT-10 (21), [4-amino-2-(prop-2-enylamino)-1,3-thiazol-5-yl]-(5-fluorothiophen-2-yl)methanone is an immunomodulatory drug that is being developed by Portage Biotech for oral administration. TT-10 exhibited a higher level of tumor growth suppression in preclinical investigations employing the 4T1 syngeneic mouse model of breast cancer than both the vehicle control and anti-PD-1 treatment groups. TT-10 therapy also resulted in a notable decrease in myeloid-derived suppressor cell (MDSC) numbers.^185,186^ The adenosine pathway is a target for cancer immunotherapy, and 2020 saw the publication of the first clinical data supporting this theory. In this trial, 68 patients with renal cell carcinoma were treated with either ciforadenant alone or with atezolizumab (PD-L1 inhibitor). Many of these patients had tumors that were primarily PD-L1-negative and were resistant to or refractory to anti-PD-1/PD-L1 antibodies. The research emphasized that in patients with resistant renal cell carcinoma, anti-PD-L1 combination therapy, and monotherapy both had antitumor efficaciousness. Compared to combination therapy, which had a median progression-free survival of 5.8 months, monotherapy had a median survival of 4.1 months. Furthermore, overall survival rates for monotherapy at 16 months and combination therapy at 25 months were higher than 69% and 90%, respectively. A_2A_ AR antagonists' efficacy in immunotherapy for various cancer types has also been shown in additional trials. The research emphasized that in patients with resistant renal cell carcinoma, anti-PD-L1 combination therapy and monotherapy both had antitumor efficaciousness. In comparison to combination therapy, which had a median progression-free survival of 5.8 months, monotherapy had a median survival of 4.1 months. A_2A_ AR antagonists' efficacy in immunotherapy for a range of cancer types has also been shown in additional trials.^187–189^

A recent Phase Ib/II trial (NCT03099161) evaluated the safety of Preladenant (22), both standalone and in combination with the anti-PD-1 drug Pembrolizumab, in patients with advanced cancers.^190^ However, the results have not yet been released.

In a Phase 1 open-label, multicenter trial, patients with advanced solid tumors will receive continuous oral administration of AZD4635 (20) for evaluation.^103,104^ Determining the maximal safe dosage of AZD4635 in conjunction with the anti-PD-L1 medication Durvalumab (NCT02740985) is the main goal of the trial.^191^ In patients with non-small cell lung cancer (NSCLC), this A_2A_ antagonist will also be evaluated for safety, tolerability, and anticancer effectiveness in combination with the anti-CD73 medication MEDI9497 and the EGFR inhibitor osimertinib (NCT03381274).^183^

There are several other high-affinity and selective adenosine A_2A_ receptor inhibitors that have been discovered with potential clinical applications. These include Etrumadenant (AB928) (23), Inupadenant (24), ANR 94 (25), CPI-444 analogue (26). A_2A_ AR selectivity has been achieved by altering xanthines at the 8-position with alkenes, especially styryl groups. One of the earliest recognised A_2A_ AR antagonists was 8-styryl-xanthine, istradefylline (27) (KW6002).^183,192,193^ The US-FDA has approved istradefylline for the treatment of Parkinson's disease.^194^ This milestone, coupled with advancing insights into the role of adenosine (ADO) in cancer biology, is anticipated to accelerate the development of ADO receptor ligands as promising anticancer agents or as adjunct therapy to existing treatments.

### 10.5 The A_2B_ receptor selective antagonists

**Fig. 12:**
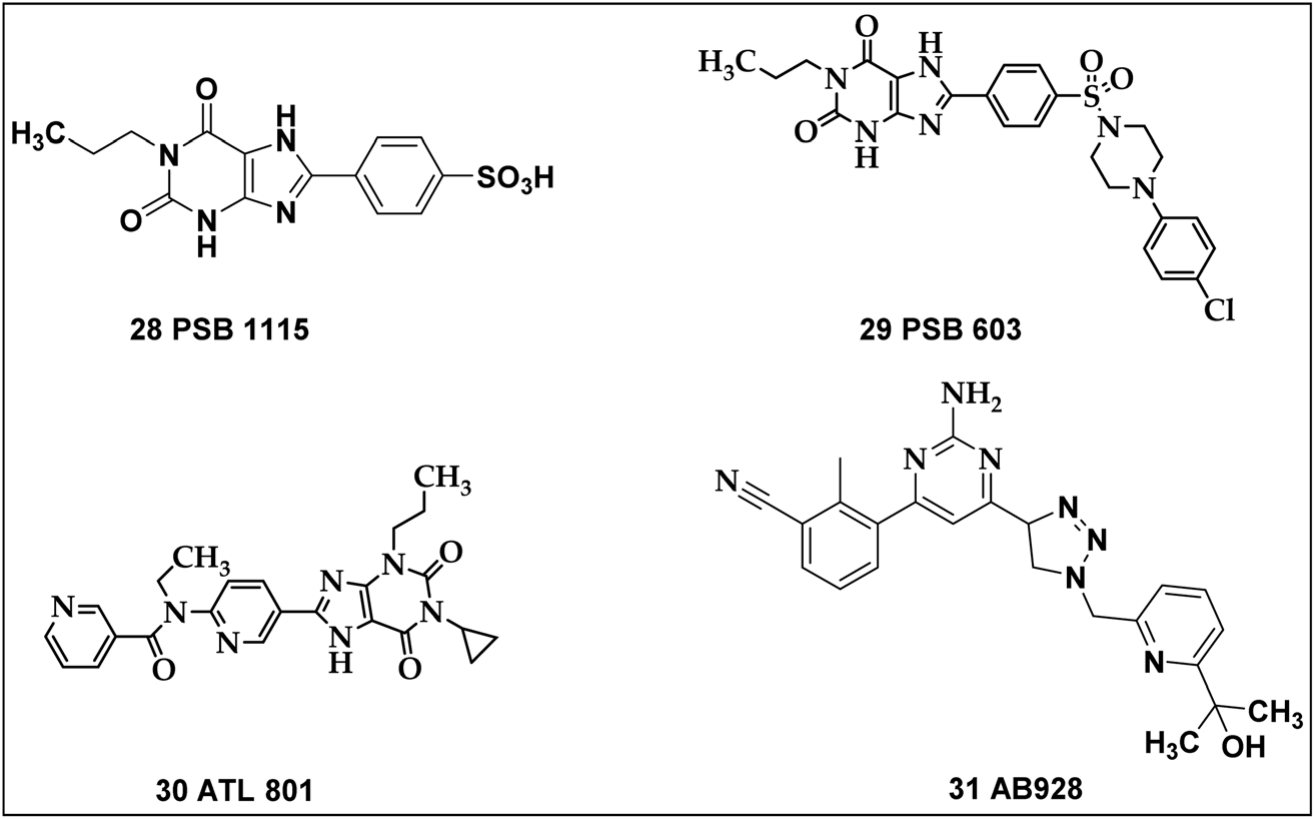
Potential A_2B_ adenosine receptor antagonists exhibiting promising anticancer properties.

The release of angiogenic factors from vascular smooth muscle, endothelial cells, and host immune cells is thought to be facilitated by the stimulation of the A_2B_ AR, which in turn aids in the formation of tumors.^145,195^ Conversely, blocking the A_2B_ AR increases the activation of dendritic cells (DC) and thus increases the synthesis of CXCL10 (C-X-C pattern chemokine 10) which is induced by IFN-γ. This chemokine contributes to the activation of lymphocytes and the induction of an angiostatic response in malignancies.^196^ Antitumor actions of several synthetic A_2B_ adenosine receptor antagonists have shown promise, as illustrated in Fig. 12.

Specific modifications of the xanthine core at the 8-position with aryl groups have been shown to confer selectivity for the A_2B_ AR. In PSB-1115 (28), modification of the xanthine core with an aryl group at the 8-position enhances receptor selectivity. Studies have demonstrated that melanoma growth can be effectively slowed by selectively inhibiting the A_2B_ AR with the antagonist “PSB1115 (4-(2,6-dioxo-1-propyl-3,7-dihydropurin-8-yl)benzenesulfonic acid)” (28). To do this, antitumor immune responses are reactivated, and the growth of myeloid-derived suppressor cells (MDSCs) within tumors is suppressed.^197^

Furthermore, studies have shown that employing the antagonist 28 targets the A_2B_ AR, which is known to play a role in suppressing the immune response within tumors. By inhibiting this receptor, the immune system can become more effective against the cancer, leading to a significant reduction in tumor growth. Compound 28, derived from xanthine, is effective in diminishing specific immune cell populations associated with tumors (*Gr-1*^+^*CD11b*^+^ cells) in melanoma. Additionally, it decreases the production of key regulatory molecules, such as interleukin-10 (IL-10) and monocyte chemoattractant protein 1 (MCP-1) that can suppress immune responses, potentially enhancing the body's ability to fight the tumor. The T cells that release cytokines like those produced by T helper 1 (Th1) cells are linked to these effects, and there are higher numbers of CD8^+^ T cells and natural killer T (NKT) cells within tumours. These outcomes imply that the ability of the A_2B_ AR antagonist 28 to minimise the infiltration of MDSCS (myeloid-derived suppressor cells) in tumours and to strengthen the anti-cancer T cell response is related to its efficacy. Furthermore, the xanthine derivative 28 has been found to drastically reduce the metastasis of B16F10 CD73^+^ malignancies by blocking the A_2B_ AR.^198^

“PSB603 (8-(4-(4-chlorophenyl)piperazide-1-sulfonyl)phenyl)-1-propylxanthine” (29), a recognized A_2B_ receptor antagonist, exhibits particularly high affinity and selectivity for the A_2B_ receptor, not only in humans but also in rodents. PSB-1115 also offers high water solubility, making it suitable for *in vivo* studies; however, its A_2B_ receptor affinity and selectivity are lower than other A_2B_ antagonists.^199^ PSB603 is being investigated in C57BL/6 mice bearing B16 melanoma to see how it affects tumour progression. The *in vivo* observations revealed a significant decrease in tumour volume, postponed tumour growth, and decreased metastasis, associated with a decrease in the regulatory T-cell population in mice. This antagonist increases CD4^+^ helper T cell and CD8^+^ cytotoxic T cell populations, which improves anti-tumor immunity in tumor-bearing animals. As a result, inhibiting the A_2B_ receptor increases the quantity of helper and cytotoxic T cells, that are necessary for the development of cancer immunity.^137,176^

ATL801 (30), an A_2B_ receptor antagonist, has been demonstrated to successfully block the growth of 4T1 mammary carcinoma and MB49 urinary bladder cancer in syngeneic animal models. It also stops breast cancer cells from metastasizing. When administered intravenously, compound 30 can elicit adaptive immunological responses in a way that is dependent on CXCR3, most likely *via* indirectly increasing the activity of dendritic cells (DCs). This process prevents the growth of tumors and helps offset the immunosuppressive effects of adenosine. These results imply that stimulating T cell activation and inhibiting formation of new blood vessel in solid tumors may be accomplished by selectively blocking the A_2B_ adenosine receptor.^200^

Lastly, it is noteworthy that a number of antagonists are presently being studied in clinical studies for different types of cancer. Notably, AB928 (31), a dual A_2A_/A_2B_ antagonist, is currently being investigated in patients with breast tumor, non-small-cell pulmonary carcinoma, and ovarian carcinoma after demonstrating encouraging findings in a Phase I clinical trial.^137,201^

### 10.6 The A_3_ receptor selective agonists

**Fig. 13:**
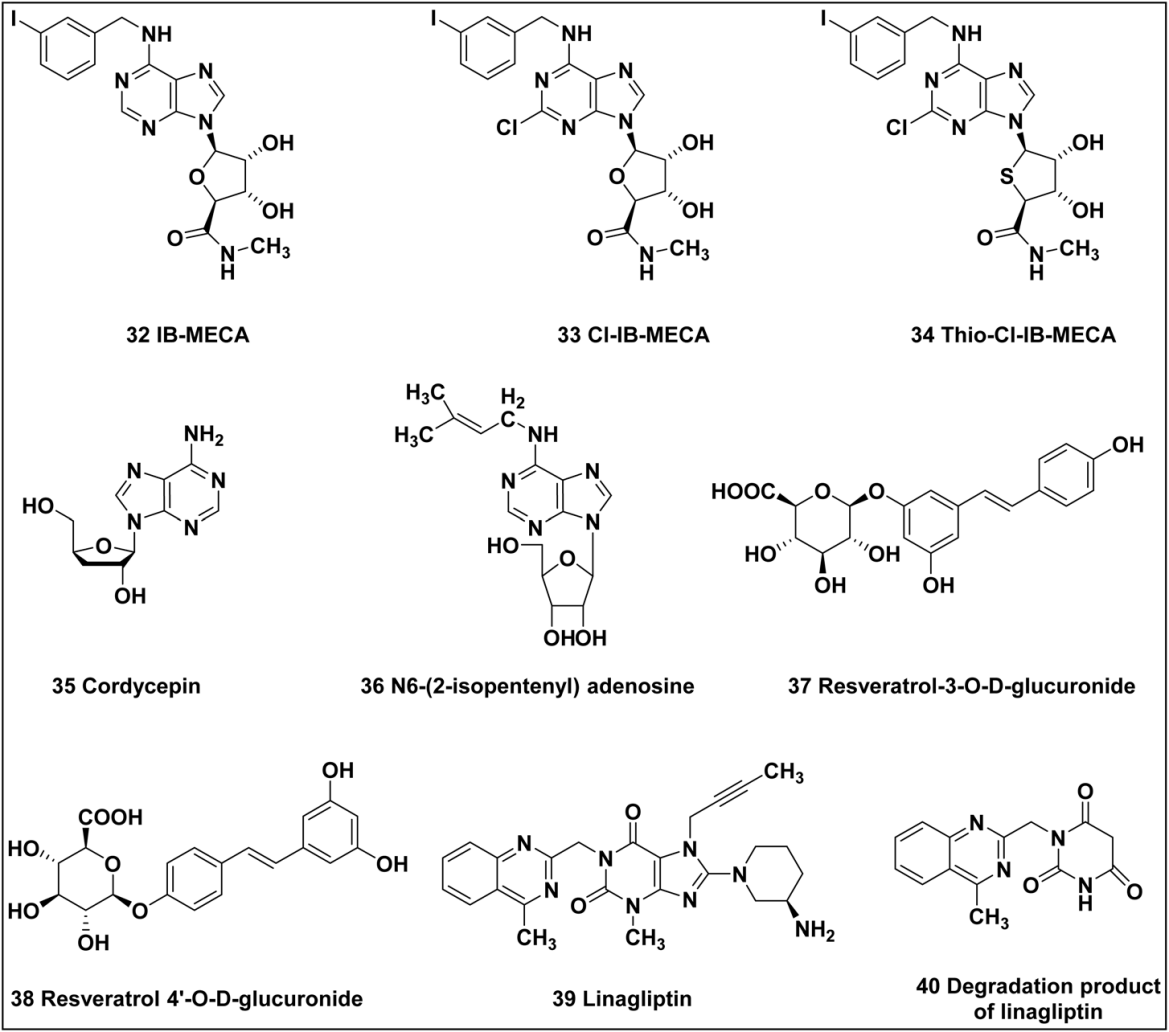
Potential A_3_ adenosine receptor agonists exhibiting promising anticancer properties.

The possibility of A_3_ receptors as a therapeutic target for the treatment of cancer is highlighted by their overexpression in malignant and inflammatory cells.^202^ Various potential A_3_ receptor agonists are illustrated in Fig. 13. Interestingly, the effects of these agonists on tumor cell proliferation differ from those on normal cell growth. Most of the A_3_ AR agonists developed to date are based on the nucleoside structure of the endogenous ligand, adenosine. The most effective enhancements in A_3_ AR potency and selectivity have been achieved through substitutions at the *N*^6^-, *C*_2_-, and 5′-positions, or through strategic combinations of these modifications.

Potent and selective A_3_ AR agonists were developed by combining *N*^6^-substitution with a 5′-uronamide group. The first A_3_ AR-selective compounds featured a 5′-*N*-alkyluronamide moiety paired with an *N*^6^-benzyl group. A key representative of this series, *N*^6^-(3-iodobenzyl)adenosine-5′-*N*-methyluronamide (IB-MECA; 32), also known as piclidenoson was discovered in 1994.^203,204^

Extensive studies have explored the effects of various substitution patterns at the *N*^6^- and *C*_2_-positions of the adenine core in 5′-*N*-alkylcarbamoyladenosine derivatives. Introducing small functional groups, such as halogens, methylamino, or thiomethyl, at the *C*_2_-position of IB-MECA has enhanced both affinity and selectivity for the A_3_ adenosine receptor (A_3_AR). This led to the development of *C*_2_-chloro-*N*^6^-(3-iodobenzyl)-5′-*N*-methylcarboxamidoadenosine (Cl-IB-MECA), a highly selective A_3_ AR agonist now considered the prototypical ligand for this receptor subtype. Cl-IB-MECA exhibits very high affinity towards rat A_3_ AR (*K*_i_ = 0.33 nM), compared to the rat A_1_ and A_2A_ receptors with *K*_i_ 820 nM and 470 nM, respectively.^205^ Both the A_3_ receptor agonists piclidenoson (IB-MECA; 32) and namodenoson (Cl-IB-MECA; 33), have a noteworthy effect even at low doses on the proliferation of tumor cells.^139,206^

When combined with 5-fluorouracil, compound 32 demonstrates more effective growth inhibition of HCT-116 human colon carcinoma cells compared to the use of 5-fluorouracil alone.^207^ Studies looking into the effects of this ligand on cells expressing estrogen receptor α (ERα) have discovered that derivative 33 quickly lowers the levels of ERα at both the mRNA and protein levels. Different kinds of breast cancer cells may experience apoptosis as a result of this action, which hinders cell proliferation.^208,209^

The synthetic A_3_ AR agonist 32, even at low nanomolar concentrations, can inhibit the growth of HCT-116 human colon carcinoma cells. Moreover, the agonist 32 has a synergistic anticancer action when coupled with 5-FU. Additionally, it reduces the myelotoxicity brought on by 5-FU, preserving normal neutrophil and white blood cell numbers. These results show that in mice with colon cancer, the A_3_ AR agonist 32 provides systemic anticancer, antimetastatic, and myeloprotective effects. It may also function as an adjuvant therapy to increase the efficiency of chemotherapy and lessen myelotoxicity.^210^

The chloro-substituted adenosine derivative 33 showed a dose-dependent reduction of Hep-3B cell proliferation at dosages of 1 and 10 nM in a xenograft animal model employing Hep-3B hepatocellular carcinoma (HCC) cells. Compound 33 effectively reduces HCC tumor growth *in vivo*, as evidenced by the differences in tumor size between the compound-treated and vehicle-treated groups after 45 days of tumor inoculation. The leading cause of this effect is the elevated production of apoptotic proteins, which are activated by the compound 33, including FasR, caspase-8, Bax, Bad, cytochrome-*c*, and caspase-3.^211^

It has been demonstrated that micromolar doses of the A_3_ AR agonist 2-chloro-*N*^6^-(3-iodobenzyl)-5′-*N*-methylcarbamoyl-4′-thioadenosine, a sulfur-containing analogue of Cl-IB-MECA,^212^ often referred to as LJ-529 or thio-Cl-IB-MECA (34), cause anti-leukemic actions in HL-60 human leukemia cell cultures. Studies on poly(ADP-ribose) polymerase (PARP) cleavage and DNA fragmentation support the apoptotic explanation for this impact.^213^ In a different investigation, it was discovered that agonist 34 inhibited the growth of breast cancer cells' tumors *in vivo* and their *in vitro* proliferation by causing apoptosis and interfering with the Wnt signaling pathway. The thioadenosine derivative 34 has been connected to the down-regulation of c-ErbB2, a critical marker for the prognosis and treatment of breast cancer, as well as the molecular mechanisms behind these effects. In both *in vitro* and *in vivo* experiments, this down-regulation is seen in SK-BR-3 breast cancer cells, suggesting that the molecule may be useful in the treatment of breast cancer.^214^ Interestingly, by acting on A_3_ AR, the herbal compound cordycepin (3′-deoxyadenosine) (35) that is extracted from the parasitic fungus *Cordyceps sinensis*, which is utilized in traditional Chinese medicine, inhibits the growth of tumor cells.^215^ At micromolar concentrations, this molecule significantly inhibited the growth of murine B16-BL6 melanoma and Lewis lung carcinoma (LCC) tumor cells. Aqueous extracts from *Cordyceps sinensis* have been investigated recently for their potential anticancer and antimetastatic effects *in vitro* using mouse melanoma B16 and LLC cells as models. These extracts showed direct cytotoxic effects at 10 and 30 μg mL^−1^, respectively. Furthermore, an *in vivo* investigation employing oral cordycepin (35) in B16-BL6 cell tumor-bearing C57BL/6Cr mice demonstrated a decrease in the main tumor weight without resulting in a reduction in body weight or systemic toxicity. With little side effects, cordycepin seems to be a potential treatment for melanoma.^216^

Comparable antiproliferative effects to Cl-IB-MECA (33) were shown by the naturally occurring anticancer nucleoside *N*^6^-(2-isopentenyl) adenosine (36), which shows strong affinity and selectivity for A_3_ AR in human and rat tumor cell lines LNCaP and N1S1.^217^

It has been demonstrated that the resveratrol glucuronides like resveratrol-3-*O*-d-glucuronide (37) and resveratrol 4′-*O*-d-glucuronide (38), with IC_50_ values ranging from 9.8 to 31 μM, suppress the development of colon cancer cells Caco-2, HCT-116, and CCL-228. In CCL-228 and Caco-2 cells, these glucuronides similarly caused a G1 phase arrest.^218^ However, the adenosine A_3_ receptor antagonist MRS1191 (48), which reversed the growth inhibition caused by these two compounds, provided direct proof that the biological activity of these drugs is mediated through A_3_ AR. Further evidence that A_3_ AR is involved in this process comes from the G1 phase arrest and cyclin D1 depletion mechanisms used to suppress cell proliferation.^170,219^

The compound 3,7-dihydro-1*H*-purine-2,6-dione, known as linagliptin (39), is an FDA-certified anti-hyperglycemic medication used primarily to manage type 2 diabetes. The principal oxidative degradation product of linagliptin (40) is a pyrimidine derivative. Both compounds, 39 and 40, have been assessed due to their capacity to harm cells, A_3_ AR binding compatibility, cAMP levels, and apoptosis-inducing capabilities. They demonstrated inhibitory effects against hepatocellular carcinoma cell lines, inducing apoptosis at the G2/M phase, increasing caspase-3 levels, and causing suppression of the A_3_ AR gene and protein expression, which was followed by an enhancement in cAMP levels. Linagliptin's quantitative *in vitro* binding affinity for A_3_ AR displays a blocking characteristic with a 37.7 nM *K*_i_ value.^220^

### 10.7 The A_3_ receptor selective antagonists

A_3_ AR antagonists have garnered attention due to their potential as cancer therapeutics and are frequently linked to anti-inflammatory properties.^221,222^

MRS1523 (41), a pyridine derivative, is currently the most commonly used selective antagonist for the rat A_3_ AR. However, its reported affinity and selectivity show some variability across different studies.^223,224^ The reported *K*_i_ values of MRS1523 for the A_3_ adenosine receptor are 43.9 nM in humans, 349 nM in mice, and 216 nM in rats.^225^

**Fig. 14:**
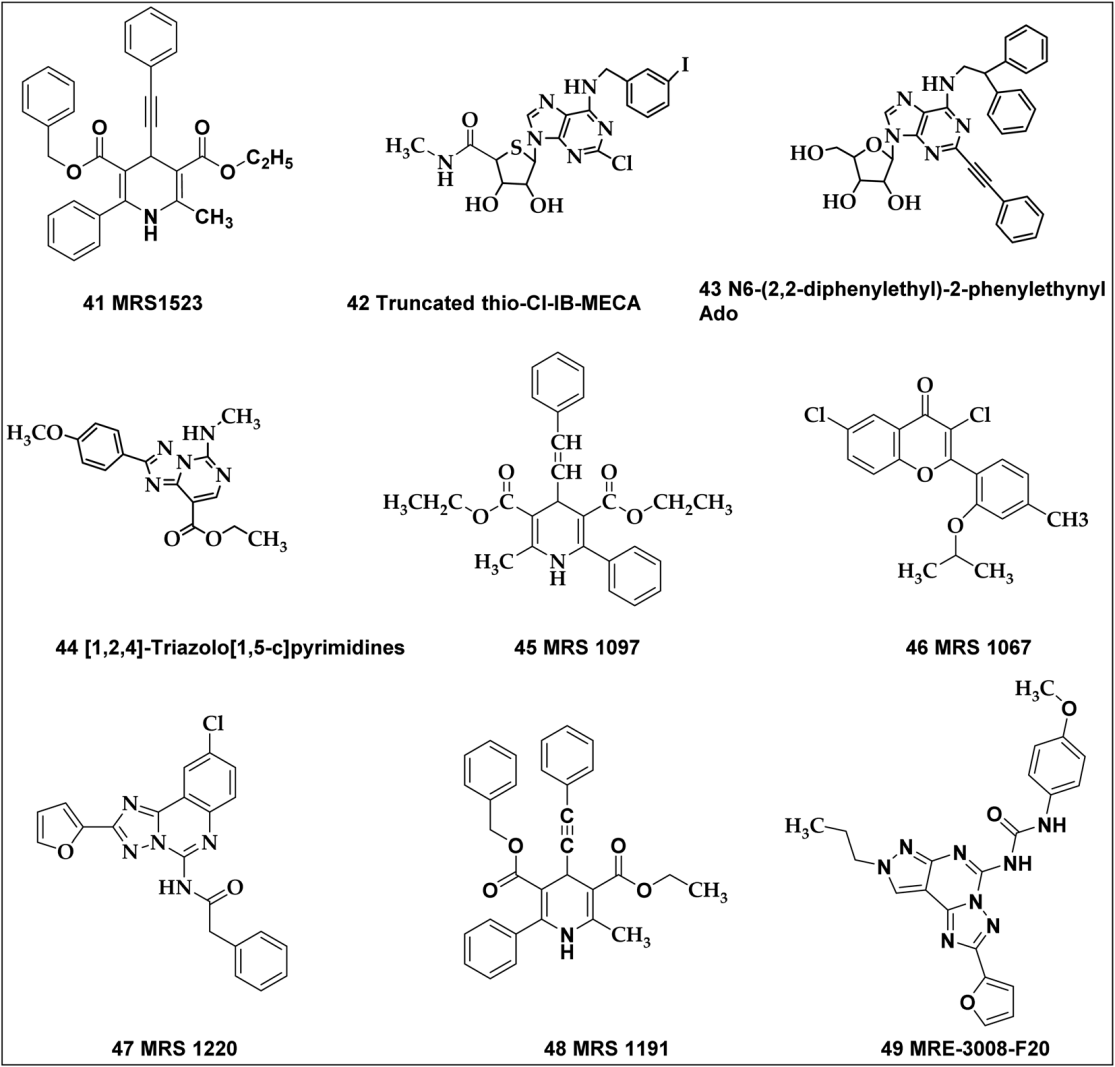
Potential A_3_ adenosine receptor antagonists exhibiting promising anticancer properties.

The antagonistic action of truncated thio-Cl-IB-MECA (42) (Fig. 14) on A_3_ AR is responsible for the inhibition of T24 human urinary bladder carcinoma cells, resulting in sub-G1 cell cycle arrest and early and late-stage cell death.^203,226^

*N* ^6^-(2,2-Diphenylethyl)-2-phenylethynylAdo (43) in particular showed the most efficacy, indicating that it has the potential to be a strong anticancer drug. The cytostatic effects seen with the A_3_ receptor blocker Cl-IB-MECA and antagonists 46 and other related compounds highlight the fact that various cellular pathways contribute to the impact of these ligands against tumors, in addition to A_3_ AR activity, which is not the only factor responsible for the anticancer consequences.^226^

Several “[1,2,4]-triazolo[1,5-*c*]pyrimidines” (44) demonstrated a strong tumor-suppressive effect on human cancer cell lines HCT16 and THP1. Compound 44 (Fig. 14), in particular, showed notable efficacy against both cell lines. To further investigate its anticancer properties, the researchers assessed its ability to penetrate the phospholipid bilayer of the cell membrane. The compound can enter cells and interact with possible target molecules, according to the Parallel Artificial Membrane Permeability Assay (PAMPA) results, which supports the theory of an as-yet-undefined mechanism.^227^

Adenosine compounds that jointly oppose A_3_ & A_2A_ ARs and have cancer immunotherapeutic efficacy are highlighted in a recent study. As mentioned earlier, these compounds suppress the immunological checkpoint activity mediated by A_2A_ AR in addition to targeting the anticancer signaling pathway triggered by A_3_ AR inhibition. This reduces aberrant immune responses.^228^

MRS 1097 (45), MRS 1067 (46), MRS 1220 (47), & MRS 1191 (48), in contrast to focusing on a specific second messenger pathway, act as antagonists of the stimulation of [^35^S]GTPγS binding produced by agonists, according to a separate study. MRS 1220 and MRS 1191, with *K*_B_ values of 1.7 nM and 92 nM, respectively, demonstrated strong selectivity for the human A_3_ AR over the human A_1_ AR in mediating effects on adenylate cyclase.^229^ These agents demonstrated high selectivity in blocking the inhibitory effects of AC mediated by human A_3_ receptors compared to those mediated by human A_1_ receptors.

Saturation binding experiments with the radiolabeled agonist [^125^I]AB-MECA (*N*^6^-(4-amino-3-iodobenzyl)adenosine-5′-N*-*methyluronamide) at cloned human brain A_3_ AR produced in HEK-293 cells demonstrated the competitiveness of MRS 1220, MRS 1191, and MRS 1067. Functional tests, such as agonist-induced suppression of AC and activation of [^35^S]GTPγS binding to related G-proteins, were used to validate their antagonistic characteristics. When it came to their actions on AC, MRS 1220 and MRS 1191, whose *K*_B_ values were 1.7 nM and 92 nM, respectively, showed strong selectivity for human A_3_ receptors as opposed to human A_1_ receptors. Additionally, MRS 1220 has shown effectiveness at reversing the A_3_ agonist-induced reduction of tumour necrosis factor-α (TNF-α) production in the human macrophage U-937 cell line.^193^

Research has explored the pre- or co-administration of pharmaceutical formulations containing high-affinity adenosine A_3_ AR antagonists like the triazine derivative MRE-3008-F20 (49), to enhance the effectiveness of chemotherapy. This includes treatments in combination with taxanes (paclitaxel), vinca alkaloids (vincristine), camptothecins (irinotecan), or antibacterial agents (doxorubicin).^230^

## 11. Clinical trials updates on adenosine receptors' modulators

Adenosine receptor antagonists are in clinical trials to investigate the treatment of various cancers. These agents block adenosine signalling to overcome tumour-induced immune suppression and boost anti-tumour immunity. Table 3 summarises key modulators under investigation, including their targets, cancer types, and combination strategies.^185,231^

**Table 3:** List of adenosine receptor modulators currently in clinical trials for treating various cancers, based on data available from ClinicalTrials.gov (https://clinicaltrials.gov/)

Compounds	Clinical trial identifier	Phase	Pharmaceutical sponsor	Patients	Description
**A**_**2A**_ **receptor antagonists**					
Ciforadenant (CPI-444)	NCT02655822	I/Ib	Corvus pharmaceuticals, Inc	Carcinoma of the renal cells and mCRPC	A selective A_2A_ AR antagonist that binds to the A_2A_ AR with a *K*_i_ value of 3.54 nM and exhibits over 50 times greater selectivity for A_2A_ receptors compared to other adenosine receptor subtypes^232^
	NCT04280328	Ib		Relapsed multiple myeloma	
	NCT03454451	I			
	NCT03237988	I		Healthy subjects	
	NCT03337698	Ib/II	Hoffmann-La Roche	Non-small cell lung cancer	
	NCT05501054	Ib/II	MD Anderson Cancer Center	Advanced renal cell carcinoma	
Taminadenant (PBF-509/NIR 178)	NCT03207867	II^a^	Novartis Pharmaceuticals II	NSCLC, carcinoma of the renal cells, pancreatic cancer, head and neck cancer, urothelial cancer, diffused large B cell lymphoma, TNBC, microsatellite stable colon cancer, melanoma, and mCRPC	A_2A_ AR antagonist^185^
	NCT03549000	I/Ib^a^		NSCLC, TNBC, pancreatic ductal adenocarcinoma, colorectal cancer microsatellite stable, ovarian cancer, carcinoma of the renal cells, and mCRPC	
	NCT04895748	I/Ib		Renal cell cancer	
	NCT02403193	I/II	Palobiofarma SL/Novartis Pharmaceuticals	Advanced NSCLC	
Preladenant	NCT03099161	Ib/II^a^	Merck Sharp & Dohme LLC	Advanced solid tumours	Strong and competitive adenosine A_2A_ AR antagonist^233^
ILB-2109	NCT05278546	Ia	Innolake Biopharm	Advanced solid tumours	A_2A_ AR antagonist
	NCT05955105			Advanced solid tumours	
AZD	NCT02740985	Ia/Ib	AstraZeneca	Advanced solid malignancies, colorectal carcinoma, mCRPC, and NSCLC	Oral A_2A_ AR antagonist (*K*_i_ value of 1.7 nM) binds to the human A_2A_ receptor and has about 30 times the selectivity for A_2A_ receptors over other adenosine receptor subtypes^234^
	NCT04089553	II		Prostate cancer and mCRPC	
	NCT03980821	I		Advanced solid malignancies	
	NCT04495179	II		Progressive mCRPC	
	NCT03381274	Ib/II	MedImmune LLC	Advanced epidermal growth factor receptor mutant NSCLC	
Inupadenant (EOS-100850/EOS-850)	NCT05060432	I/II	iTeosTherapeutics	Advanced solid tumors	Inupadenant is a highly selective oral A_2A_ AR antagonist that is blood–brain barrier insensitive^235^
	NCT05403385	II		Advanced or metastatic non-small cell lung cancer	
	NCT05117177	I		Advanced lung non-small cell carcinoma	
TT-10	NCT04969315	I/II	Portage Biotech	NSCLC, mCRPC, renal cell cancer, and head and neck cancer	Potent and selective antagonists of A_2A_ AR^185,236^

**Antagonists of both A**_**2A**_ **and A**_**2B**_ **receptors**					
Etrumadenant (AB928)	NCT03720678	I/Ib	Arcus Biosciences	Gastroesophageal cancer and colorectal cancer	A_2A_ AR and A_2B_ AR have *K*_d_ values of 1.4 nM and 2 nM, respectively, for this new dual-active A_2A_/A_2B_ AR antagonist^201^
	NCT04381832	Ib/II		mCRPC	
	NCT04892875	Ib	Vanderbilt-Ingram Cancer Center	Locally advanced head and neck cancers	
	NCT05024097	I/II	Weill Medical College of Cornell University	Rectal cancer	
	NCT05886634	II	Memorial Sloan Kettering Cancer Center	Advanced dedifferentiated liposarcoma	
	NCT04660812	Ib/II	Arcus Biosciences	Metastatic colorectal cancer	
	NCT05177770	II	Surface oncology	mCRPC	
M1069	NCT05198349	I^a^	EMD Serono Research & Development Institute	Locally advanced unresectable solid tumors	A_2A_/A_2B_ AR antagonist

**A**_**3**_ **receptor agonists**					
Namodenoson/Cl-IB-MECA	NCT00790218	I-II	Can-Fite BioPharma Ltd	Hepatocellular carcinoma	Only an A_3_ AR agonist with such high affinity has been found to date
	NCT02128958	II		Hepatocellular carcinoma	

a Study terminated.

## 12 Summary and future perspectives

AR activation affects immune suppression, angiogenesis, development of tumours, cell proliferation, and metastasis, all of which are essential to the progression of cancer. In the TME, extracellular adenosine concentration is much higher than normal. This elevated level stimulates adenosine receptors, which then trigger various biological responses that can inhibit cancer cell growth and enhance the immune system's ability to target tumors. The existing literature on AR subtypes indicates that each subtype is significantly involved in cancer, supported by findings from both *in vitro* and *in vivo* research. Because of this, all four AR subtypes are thought to be viable targets for the development of innovative therapeutic approaches in the treatment of cancer. Clinical trials focusing on Parkinson's disease have assessed the safety of A_2A_ AR antagonists. The role of A_2B_ ARs in tumors is not fully understood. While these receptors may aid in tumour growth by releasing substances that encourage the formation of angiogenesis, they might also send signaling that blocks the growth of cancer cells. Their feasibility as therapeutic targets in the therapy of cancer is complicated by this contradiction. Medications that target A_2B_ adenosine receptors (ARs) by either activating or inhibiting them have proven to be more effective in treating cancer. Meanwhile, synthetic drugs that activate A_3_ ARs have been effective in slowing down cancer cell growth and promoting programmed apoptosis on various types of cancer. This effectiveness has been validated through laboratory studies and experiments on animals, highlighting the potential of these receptors as therapeutic targets in cancer treatment. Specifically, activating the A_3_ receptor or blocking the A_1_, A_2A_, and A_2B_ receptors could help shift the tumor environment from one that supports cancer growth to one that inhibits it, enhancing the body's ability to fight cancer. In this regard, numerous modulators targeting adenosine receptors (A_1_, A_2A_, A_2B_, and A_3_), including preladenant, tozadenant, CPI444, NIR 178, PBF-509, M1069, Inupadenant, and Cl-IB-MECA, are in advanced clinical stages. However, many have failed for various reasons,^185^ including unsatisfactory pharmacokinetic properties. Moreover, developing adenosine receptor agonists or antagonists with high target selectivity and potency remains a significant challenge due to the body's widespread presence of adenosine receptors,^31^ raising concerns about off-target effects and limited efficacy. Moreover, data on the long-term impact of AR blockade, including tumour resistance and compensatory GPCR signalling, remain unclear. Even clinical-stage compounds often lack complete optimisation for durable and selective anti-tumour activity. These gaps emphasise the need for improved mechanistic understanding and more targeted drug development strategies.^22^

Synthetic agonist activation of the A_3_ AR decreases cell proliferation and enhances apoptosis in a range of cancer cells, as demonstrated by both *in vitro* and *in vivo* models. These agonists have already been shown in preclinical and Phase I/II trials to be safe and generally well-tolerated in human patients. However, a couple of studies revealed that A_3_ AR antagonists may also be a viable therapeutic approach for cancer by preventing hypoxia-induced rises in HIF-1α and decreasing angiogenesis and cell invasion in the tumor microenvironment. The A_2A_ receptor activation contributes to immune system suppression in the tumour microenvironment (TME) *via* cAMP signalling, making it easier for cancer to evade the immune system. Interestingly, the enzyme that changes AMP into adenosine, CD39 and CD73 are highly expressed in various TME cell types and contribute to this immune suppressive effect. Recent studies have identified CD39, CD73, and A_2A_ AR as promising targets for boosting antitumor immunity. Monoclonal antibodies and small-molecule inhibitors targeting the CD39/CD73/A_2A_ AR axis are currently being tested in clinical trials, both as standalone treatments and in combination with anti-PD-1/PD-L1 immunotherapies.^237^

Results indicate that the antitumor immune response is enhanced when these antibodies are combined with A_2A_ AR antagonists, potentially providing an impactful therapeutic strategy for cancer treatment.^170,185^

Clinical trials involving A_2A_ antagonists and A_3_ agonists will also take some time to conclude which therapy approach is more promising for cancer. The two receptors do have different biological effects: A_2A_ receptor signalling decreases immune cell antitumor activity, whereas A_3_ receptor signalling enhances immune function while inhibiting tumour growth. Thus, drugs that activate A_3_ adenosine receptors and block A_2A_ receptors are being explored as a novel approach for cancer treatment. This strategy aims to enhance the body's anti-tumour response by leveraging the beneficial effects of A_3_ receptor activation while reducing the tumour-supportive signals associated with A_2A_ receptor activation. As a result, this combination could potentially improve therapeutic outcomes for cancer patients. However, they are still in the early stages of development and must overcome several critical challenges, including stability, bioavailability, toxicity, and safety profile studies.^238^

## Abbreviations

5-AMP: 5-Adenosine monophosphate
A_1_ AR: A_1_ adenosine receptor
A_2A_ AR: A_2A_ adenosine receptor
A_2B_ AR: A_2B_ adenosine receptor
A_3_ AR: A_3_ adenosine receptor
AC: Adenylate cyclase
ADA: Adenosine deaminase
ADP: Adenosine diphosphate
AK: Adenosine kinase
AK-T cell: Activated T cell
AMP: Adenosine monophosphate
ARs: Adenosine receptors
ATP: Adenosine triphosphate
Ca^2+^: Calcium channels
cAMP: Cyclic adenosine monophosphate
CNS: Central nervous system
CNTs: Concentrative nucleoside transporters
CPA: Cyclopentyl adenosine
CREB: Camp-response element binding protein
CTLs: Cytotoxic T lymphocytes
DAG: Diacylglycerol
ecto-PDE: Ecto-phosphodiesterase
ENTs: Equilibrative nucleoside transporters
ERK1/2: Extracellular signal-regulated kinases
FoxP3: Forkhead box P3
GDP: Guanosine diphosphate
GFR: Glomerular filtration rate
G_i_/G_o_: Inhibitory G-proteins
GPCRs: G protein-coupled receptors
G_s_: Stimulatory G-proteins
G_s_/G_olf_: Stimulatory G-proteins
GSK-3β: Glycogen synthase kinase -3β
GTP: Guanosine triphosphate
IMPDH: Inosine monophosphate dehydrogenase
IP3: Inositol triphosphate
JNK: C-Jun N-terminal kinases
K^+^: Potassium channels
LAK Cell: Lymphokine-activated killer cells
MAPKs: Mitogen-activated protein kinase
MMP2: Matrix metalloproteinase 2
NK: Natural killer
PD-1: Programmed cell death protein 1
PD-L1: Programmed death-ligand 1
PGs: Prostaglandins
PIP2: Phosphatidylinositol 4,5-bisphosphate
PKA: Protein kinase A
PLC: Phospholipase C
PLC-β: Phospholipase C-beta
PNP: Purine nucleoside phosphorylase
RhoA: Ras homolog family member A
ROS: Reactive oxygen species
SAH: *S*-Adenosyl-homocysteine
SAHH: *S*-Adenosyl-homocysteine hydrolase
SAMe: *S*-Adenosylmethionine
TGF-β: Transforming growth factor-beta
TME: Tumor microenvironment
VEGFs: Vascular endothelial growth factors

## Conflicts of interest

The authors declare no conflicts of interest.

## Data Availability

No primary research results, software or code have been included and no new data were generated or analysed as part of this review.

## References

[cit1] Sachdeva S., Gupta M. (2013). Saudi Pharm. J..

[cit2] Layland J., Carrick D., Lee M., Oldroyd K., Berry C. (2014). JACC Cardiovasc. Interv..

[cit3] Bar-Yehuda S., Barer F., Volisson L., Fishman P. (2001). Neoplasia.

[cit4] Newby A. C. (1984). Trends Biochem. Sci..

[cit5] de Lera Ruiz M., Lim Y.-H., Zheng J. (2014). J. Med. Chem..

[cit6] Bahreyni A., Khazaei M., Rajabian M., Ryzhikov M., Avan A., Hassanian S. M. (2018). J. Pharm. Pharmacol..

[cit7] Gessi S., Merighi S., Sacchetto V., Simioni C., Borea P. A. (2011). Biochim. Biophys. Acta, Biomembr..

[cit8] Bahreyni A., Samani S. S., Rahmani F., Behnam-Rassouli R., Khazaei M., Ryzhikov M., Parizadeh M. R., Avan A., Hassanian S. M. (2018). J. Cell. Physiol..

[cit9] Borea P. A., Gessi S., Merighi S., Varani K. (2016). Trends Pharmacol. Sci..

[cit10] Borea P. A., Gessi S., Merighi S., Vincenzi F., Varani K. (2018). Physiol. Rev..

[cit11] Kumar P. and Deb P. K., Frontiers in Pharmacology of Neurotransmitters, Springer, 2020

[cit12] Borah P., Deka S., Mailavaram R. P., Deb P. K. (2019). Curr. Pharm. Des..

[cit13] Gessi S., Varani K., Merighi S., Cattabriga E., Iannotta V., Leung E., Baraldi P. G., Borea P. A. (2002). Mol. Pharmacol..

[cit14] Antonioli L., Blandizzi C., Pacher P., Haskó G. (2013). Nat. Rev. Cancer.

[cit15] Jadidi-Niaragh F. (2019). Immunotherapy.

[cit16] Beavis P. A., Stagg J., Darcy P. K., Smyth M. J. (2012). Trends Immunol..

[cit17] Decking U. K. M., Schlieper G., Kroll K., Schrader J. (1997). Circ. Res..

[cit18] Antonioli L., Colucci R., La Motta C., Tuccori M., Awwad O., Da Settimo F., Blandizzi C., Fornai M. (2012). Curr. Drug Targets.

[cit19] Allard B., Pommey S., Smyth M. J., Stagg J. (2013). Clin. Cancer Res..

[cit20] Deb P. K. (2019). Curr. Pharm. Des..

[cit21] Deb P. K. (2019). Curr. Pharm. Des..

[cit22] Chandrasekaran B., Samarneh S., Jaber A. M. Y., Kassab G., Agrawal N. (2019). Curr. Pharm. Des..

[cit23] Deb P. K., Kokaz S. F., Abed S. N., Chandrasekaran B., Hourani W., Jaber A. Y., Mailavaram R. P., Kumar P., Venugopala K. N. (2020). Front. Pharmacol..

[cit24] Zimmermann H. (2000). Naunyn-Schmiedeberg’s Arch. Pharmacol..

[cit25] Hay C. M., Sult E., Huang Q., Mulgrew K., Fuhrmann S. R., McGlinchey K. A., Hammond S. A., Rothstein R., Rios-Doria J., Poon E. (2016). Oncoimmunology.

[cit26] Jadidi-Niaragh F., Atyabi F., Rastegari A., Kheshtchin N., Arab S., Hassannia H., Ajami M., Mirsanei Z., Habibi S., Masoumi F. (2017). J. Controlled Release.

[cit27] Jacobson K. A., Gao Z.-G. (2006). Nat. Rev. Drug Discovery.

[cit28] Burnstock G. (2017). Front. Pharmacol..

[cit29] Müller C. E., Jacobson K. A. (2011). Biochim. Biophys. Acta, Biomembr..

[cit30] Fredholm B. B. (2010). Exp. Cell Res..

[cit31] Chen J.-F., Eltzschig H. K., Fredholm B. B. (2013). Nat. Rev. Drug Discovery.

[cit32] Varani K., Vincenzi F., Merighi S., Gessi S., Borea P. A. (2017). Protein Rev..

[cit33] Deb P. K., Deka S., Borah P., Abed S. N., Klotz K.-N. (2019). Curr. Pharm. Des..

[cit34] Dhalla A. K. , Chisholm J. W., Reaven G. M. and Belardinelli L., A_1_ Adenosine Receptor: Role in Diabetes and Obesity, Adenosine Receptors in Health and Disease, 2009, pp. 271–295 10.1007/978-3-540-89615-9_9 19639285

[cit35] Merighi S., Borea P. A., Gessi S. (2015). Pharmacol. Res..

[cit36] Ciruela F., Albergaria C., Soriano A., Cuffí L., Carbonell L., Sánchez S., Gandía J., Fernández-Dueñas V. (2010). Biochim. Biophys. Acta, Biomembr..

[cit37] BB F. (2001). Biochem. Pharmacol..

[cit38] Borea P. A., Varani K., Vincenzi F., Baraldi P. G., Tabrizi M. A., Merighi S., Gessi S. (2015). Pharmacol. Rev..

[cit39] Chen J.-F. and Mori A., Adenosine A2A Receptor Antagonists, Elsevier, 2023, vol. 170

[cit40] Fredholm B. B., Irenius E., Kull B., Schulte G. (2001). Biochem. Pharmacol..

[cit41] Raman M., Chen W., Cobb M. H. (2007). Oncogene.

[cit42] Goldsmith Z. G., Dhanasekaran D. N. (2007). Oncogene.

[cit43] Seino S., Shibasaki T. (2005). Physiol. Rev..

[cit44] Poulsen S.-A., Quinn R. J. (1998). Bioorg. Med. Chem..

[cit45] Offermanns S., Simon M. I. (1995). J. Biol. Chem..

[cit46] Fresco P., Diniz C., Gonçalves J. (2004). Cardiovasc. Res..

[cit47] Sperlágh B., Sylvester Vizi E. (2011). Curr. Top. Med. Chem..

[cit48] Godinho R. O., Duarte T., Pacini E. S. A. (2015). Front. Pharmacol.

[cit49] Peleli M., Fredholm B. B., Sobrevia L., Carlström M. (2017). Mol. Aspects Med..

[cit50] Fried N. T., Elliott M. B., Oshinsky M. L. (2017). Brain Sci..

[cit51] Deussen A., Schmiedebergs N. (2000). Arch. Pharmacol..

[cit52] Jacobson K. A., Trivedi B. K., Churchill P. C., Williams M. (1991). Biochem. Pharmacol..

[cit53] Sheth S., Brito R., Mukherjea D., Rybak L. P., Ramkumar V. (2014). Int. J. Mol. Sci..

[cit54] Stumpe T., Schrader J. (1997). Am. J. Physiol.: Heart Circ. Physiol..

[cit55] Boison D. (2013). Pharmacol. Rev..

[cit56] Gracia E., Farré D., Cortés A., Ferrer-Costa C., Orozco M., Mallol J., Lluís C., Canela E. I., McCormick P. J., Franco R. (2013). FASEB J..

[cit57] Pacheco R., Martinez-Navio J. M., Lejeune M., Climent N., Oliva H., Gatell J. M., Gallart T., Mallol J., Lluis C., Franco R. (2005). Proc. Natl. Acad. Sci. U. S. A..

[cit58] Cacciari B., Spalluto G., Federico S. (2018). Mini-Rev. Med. Chem..

[cit59] López-Cruz L., Salamone J. D., Correa M. (2018). Front. Pharmacol.

[cit60] Ferre S., Quiroz C., Woods A. S., Cunha R., Popoli P., Ciruela F., Lluis C., Franco R., Azdad K., Schiffmann S. N. (2008). Curr. Pharm. Des..

[cit61] Holst S. C., Landolt H.-P. (2015). Curr. Sleep Med. Rep..

[cit62] Ballesteros-Yáñez I., Castillo C. A., Merighi S., Gessi S. (2018). Front. Pharmacol.

[cit63] Fredholm B. B., Arslan G., Halldner L., Kull B., Schulte G., Wasserman W., Schmiedebergs N. (2000). Arch. Pharmacol..

[cit64] Ellis M. J., Lindon A. C., Flint K. J., Jones N. C., Goodbourn S. (1995). Mol. Endocrinol..

[cit65] Löffler I., Grün M., Böhmer F. D., Rubio I. (2008). BMC Cancer.

[cit66] Sakamoto K. M., Frank D. A. (2009). Clin. Cancer Res..

[cit67] Boettcher M., Lawson A., Ladenburger V., Fredebohm J., Wolf J., Hoheisel J. D., Frezza C., Shlomi T. (2014). BMC Genomics.

[cit68] Yu S.-J., Yu J.-K., Ge W.-T., Hu H.-G., Yuan Y., Zheng S. (2011). World J. Gastroenterol..

[cit69] Hong S.-H., Goh S.-H., Lee S. J., Hwang J.-A., Lee J., Choi I.-J., Seo H., Park J.-H., Suzuki H., Yamamoto E. (2013). Oncotarget.

[cit70] Mohamed R. A., Agha A. M., Abdel-Rahman A. A., Nassar N. N. (2016). Neuroscience.

[cit71] Preti D., Baraldi P. G., Moorman A. R., Borea P. A., Varani K. (2015). Med. Res. Rev..

[cit72] Schulte G., Fredholm B. B. (2003). Cell Signalling.

[cit73] Loi S., Dushyanthen S., Beavis P. A., Salgado R., Denkert C., Savas P., Combs S., Rimm D. L., Giltnane J. M., V Estrada M. (2016). Clin. Cancer Res..

[cit74] Fishman P., Bar-Yehuda S., Madi L., Cohn I. (2002). Anticancer Drugs.

[cit75] Vaupel P., Kallinowski F., Okunieff P. (1989). Cancer Res..

[cit76] Multhoff G. and Vaupel P., Oxyg. Transp. to Tissue XLI, 2020, pp. 131–143

[cit77] V Sitkovsky M., Lukashev D., Apasov S., Kojima H., Koshiba M., Caldwell C., Ohta A., Thiel M. (2004). Annu. Rev. Immunol..

[cit78] Sitkovsky M., Lukashev D. (2005). Nat. Rev. Immunol..

[cit79] Kazemi M. H., Raoofi Mohseni S., Hojjat-Farsangi M., Anvari E., Ghalamfarsa G., Mohammadi H., Jadidi-Niaragh F. (2018). J. Cell. Physiol..

[cit80] Blay J., White T. D., Hoskin D. W. (1997). Cancer Res..

[cit81] Deussen A., Stappert M., Schäfer S., Kelm M. (1999). Circulation.

[cit82] Hoskin D. W., Reynolds T., Blay J. (1994). Cancer Immunol., Immunother..

[cit83] Hoskin D. W., Reynolds T., Blay J. (1994). Int. J. Cancer.

[cit84] Hoskin D. W., Reynolds T., Blay J. (1994). Cell. Immunol..

[cit85] Hoskin D. W., Butler J. J., Drapeau D., Haeryfar S. M. M., Blay J. (2002). Int. J. Cancer.

[cit86] MacKenzie W. M., Hoskin D. W., Blay J. (2002). Exp. Cell Res..

[cit87] Burnstock G. (2002). Arterioscler., Thromb., Vasc. Biol..

[cit88] Van Daele P., Van Coevorden A., Roger P. P., Boeynaems J.-M. (1992). Circ. Res..

[cit89] Ethier M. F., Chander V., Dobson Jr J. G. (1993). Am. J. Physiol.: Heart Circ. Physiol..

[cit90] Lutty G. A., Mathews M. K., Merges C., McLeod D. S. (1998). Curr. Eye Res..

[cit91] Testa U., Castelli G., Pelosi E. (2019). Medicines.

[cit92] Basheer R., Arrigoni E., Thatte H. S., Greene R. W., Ambudkar I. S., McCarley R. W. (2002). J. Neurosci..

[cit93] Kirsch G. E., Codina J., Birnbaumer L., Brown A. M. (1990). Am. J. Physiol.: Heart Circ. Physiol..

[cit94] Kunduri S. S., Dick G. M., Nayeem M. A., Mustafa S. J. Physiol. Rep..

[cit95] Shaban M., Smith R. A., Stone T. W. (1995). Cell Proliferation.

[cit96] Synowitz M., Glass R., Färber K., Markovic D., Kronenberg G., Herrmann K., Schnermann J., Nolte C., van Rooijen N., Kiwit J. (2006). Cancer Res..

[cit97] Chen Z., Han F., Du Y., Shi H., Zhou W. (2023). Signal Transduction Targeted Ther..

[cit98] Haskó G., Pacher P., Vizi E. S., Illes P. (2005). Trends Pharmacol. Sci..

[cit99] Lv B., Wang Y., Ma D., Cheng W., Liu J., Yong T., Chen H., Wang C. (2022). Front. Immunol..

[cit100] Takagi H., King G. L., Robinson G. S., Ferrara N., Aiello L. P. (1996). Invest. Ophthalmol. Visual Sci..

[cit101] Venugopala K. N., Buccioni M. (2024). Molecules.

[cit102] Sek K., Mølck C., Stewart G. D., Kats L., Darcy P. K., Beavis P. A. (2018). Int. J. Mol. Sci..

[cit103] Gebicke-Haerter P. J., Christoffel F., Timmer J., Northoff H., Berger M., Van Calker D. (1996). Neurochem. Int..

[cit104] Gorain B., Choudhury H., Yee G. S., Bhattamisra S. K. (2019). Curr. Pharm. Des..

[cit105] Kaur T., Borse V., Sheth S., Sheehan K., Ghosh S., Tupal S., Jajoo S., Mukherjea D., Rybak L. P., Ramkumar V. (2016). J. Neurosci..

[cit106] Ghiringhelli F., Bruchard M., Chalmin F., Rébé C. (2012). BioMed Res. Int..

[cit107] Mirza A., Basso A., Black S., Malkowski M., Kwee L., Patcher J. A., Lachowicz J. E., Wang Y., Liu S. (2005). Cancer Biol. Ther..

[cit108] Zhou Y., Tong L., Chu X., Deng F., Tang J., Tang Y., Dai Y. (2017). Cell. Physiol. Biochem..

[cit109] Young A., Mittal D., Stagg J., Smyth M. J. (2014). Cancer Discovery.

[cit110] Yu F., Zhu C., Xie Q., Wang Y. (2020). J.
Med. Chem..

[cit111] Merighi S., Mirandola P., Varani K., Gessi S., Leung E., Baraldi P. G., Tabrizi M. A., Borea P. A. (2003). Pharmacol. Ther..

[cit112] Muller-Haegele S., Muller L., Whiteside T. L. (2014). Expert Rev. Clin. Immunol..

[cit113] Gessi S., Bencivenni S., Battistello E., Vincenzi F., Colotta V., Catarzi D., Varano F., Merighi S., Borea P. A., Varani K. (2017). Front. Pharmacol..

[cit114] Bova V., Filippone A., Casili G., Lanza M., Campolo M., Capra A. P., Repici A., Crupi L., Motta G., Colarossi C. (2022). Cancers.

[cit115] Xing J., Zhang J., Wang J. (2023). Int. J. Mol. Sci..

[cit116] Hatfield S. M., Sitkovsky M. (2016). Curr. Opin. Pharmacol..

[cit117] Rivkees S. A., Reppert S. M. (1992). Mol. Endocrinol..

[cit118] Pierce K. D., Furlong T. J., Selbie L. A., Shine J. (1992). Biochem. Biophys. Res. Commun..

[cit119] Schulte G., Fredholm B. B. (2003). Exp. Cell Res..

[cit120] Fredholm B. B., IJzerman A. P., Jacobson K. A., Linden J., Müller C. E. (2011). Pharmacol. Rev..

[cit121] Gao Z.-G., Jacobson K. A. (2019). Int. J. Mol. Sci..

[cit122] Robichaux III W. G., Cheng X. (2018). Physiol. Rev..

[cit123] Fang Y., Olah M. E. (2007). J. Pharmacol. Exp. Ther..

[cit124] Mayer P., Hinze A. V., Harst A., von Kügelgen I. (2011). Cardiovasc. Res..

[cit125] Merighi S., Benini A., Mirandola P., Gessi S., Varani K., Leung E., Maclennan S., Baraldi P. G., Borea P. A. (2007). Mol. Pharmacol..

[cit126] Vecchio E. A., White P. J., May L. T. (2019). Pharmacol. Ther..

[cit127] Martin A. F. , Molecular Analysis of A2A Adenosine Receptor Regulation of NF-κb-dependent Inflammatory Responses, University of Glasgow, United Kingdom, 2004

[cit128] Allard D., Turcotte M., Stagg J. (2017). Immunol. Cell Biol..

[cit129] Phosri S., Arieyawong A., Bunrukchai K., Parichatikanond W., Nishimura A., Nishida M., Mangmool S. (2017). Front. Pharmacol..

[cit130] Phosri S., Bunrukchai K., Parichatikanond W., Sato V. H., Mangmool S. (2018). Purinergic Signalling.

[cit131] Koscsó B., Csóka B., Selmeczy Z., Himer L., Pacher P., Virág L., Haskó G. (2012). J. Immunol..

[cit132] Merighi S., Bencivenni S., Vincenzi F., Varani K., Borea P. A., Gessi S. (2017). Pharmacol. Res..

[cit133] Chin A., Svejda B., Gustafsson B. I., Granlund A. B., Sandvik A. K., Timberlake A., Sumpio B., Pfragner R., Modlin I. M., Kidd M. (2012). Am. J. Physiol. Liver Physiol..

[cit134] Yang X., Xin W., Yang X., Kuno A., Rich T. C., V Cohen M., Downey J. M. (2011). Br. J. Pharmacol..

[cit135] Sun Y., Huang P. (2016). Front. Chem..

[cit136] Wei W., Du C., Lv J., Zhao G., Li Z., Wu Z., Haskó G., Xie X. (2013). J. Immunol..

[cit137] Cekic C., Sag D., Li Y., Theodorescu D., Strieter R. M., Linden J. (2012). J. Immunol..

[cit138] Fishman P., Bar-Yehuda S., Ohana G., Barer F., Ochaion A., Erlanger A., Madi L. (2004). Oncogene.

[cit139] Fishman P., Bar-Yehuda S., Liang B. T., Jacobson K. A. (2012). Drug Discovery Today.

[cit140] Suh B., Kim T., Lee J., Seong J., Kim K. (2001). Br. J. Pharmacol..

[cit141] Gessi S., Cattabriga E., Avitabile A., Gafa’ R., Lanza G., Cavazzini L., Bianchi N., Gambari R., Feo C., Liboni A. (2004). Clin. Cancer Res..

[cit142] Bar-Yehuda S., Stemmer S. M., Madi L., Castel D., Ochaion A., Cohen S., Barer F., Zabutti A., Perez-Liz G., Del Valle L. (2008). Int. J. Oncol..

[cit143] Madi L., Ochaion A., Rath-Wolfson L., Bar-Yehuda S., Erlanger A., Ohana G., Harish A., Merimski O., Barer F., Fishman P. (2004). Clin. Cancer Res..

[cit144] Morello S., Petrella A., Festa M., Popolo A., Monaco M., Vuttariello E., Chiappetta G., Parente L., Pinto A. (2008). Cancer Biol. Ther..

[cit145] Merighi S., Mirandola P., Milani D., Varani K., Gessi S., Klotz K.-N., Leung E., Baraldi P. G., Borea P. A. (2002). J. Invest. Dermatol..

[cit146] Azoulay-Alfaguter I., Elya R., Avrahami L., Katz A., Eldar-Finkelman H. (2015). Oncogene.

[cit147] Jajoo S., Mukherjea D., Watabe K., Ramkumar V. (2009). Neoplasia.

[cit148] Atkinson M. R., Townsend-Nicholson A., Nicholl J. K., Sutherland G. R., Schofield P. R. (1997). Neurosci. Res..

[cit149] Yan L., Burbiel J. C., Maaß A., Müller C. E. (2003). Expert Opin. Emerging Drugs.

[cit150] Gao Z.-G., Jacobson K. A. (2007). Expert Opin. Emerging Drugs.

[cit151] Gao Z.-G., Jacobson K. A. (2011). Expert Opin. Emerging Drugs.

[cit152] Bruns R. F. (1981). Biochem. Pharmacol..

[cit153] Trivedi B. K. , Bridges A. J. and Bruns R. F., in Adenosine and Adenosine Receptors, Springer, 1990, pp. 57–103

[cit154] Schenone S., Brullo C., Musumeci F., Bruno O., Botta M. (2010). Curr. Top. Med. Chem..

[cit155] Klotz K.-N., Schmiedebergs N. (2000). Arch. Pharmacol..

[cit156] Gao Z., Jacobson K. A. (2002). Eur. J. Pharmacol..

[cit157] Muller C. E. (2000). Curr. Med. Chem..

[cit158] Gao Z.-G., Blaustein J. B., Gross A. S., Melman N., Jacobson K. A. (2003). Biochem. Pharmacol..

[cit159] Jacobson K. A., Van Galen P. J. M., Williams M. (1992). J. Med. Chem..

[cit160] Carlin J. L., Jain S., Gizewski E., Wan T. C., Tosh D. K., Xiao C., Auchampach J. A., Jacobson K. A., Gavrilova O., Reitman M. L. (2017). Neuropharmacology.

[cit161] Franchetti P., Cappellacci L., Vita P., Petrelli R., Lavecchia A., Kachler S., Klotz K.-N., Marabese I., Luongo L., Maione S. (2009). J. Med. Chem..

[cit162] Jacobson M. A. (2002). Expert Opin. Ther. Pat..

[cit163] Nell P. G., Albrecht-Küpper B. (2009). Prog. Med. Chem..

[cit164] Blay J. , Encycl. Cancer, SpringerBerlin Heidelb., 2012, 49–52

[cit165] Etique N., Grillier-Vuissoz I., Lecomte J., Flament S. (2009). Oncol. Rep..

[cit166] Perez-Aso M., Mediero A., Low Y. C., Levine J., Cronstein B. N. (2015). FASEB J..

[cit167] Ohta A., Gorelik E., Prasad S. J., Ronchese F., Lukashev D., Wong M. K. K., Huang X., Caldwell S., Liu K., Smith P. (2006). Proc. Natl. Acad. Sci. U. S. A..

[cit168] Muller C. E., Ferré S. (2010). Front. CNS Drug Discovery.

[cit169] Shah U., Hodgson R. (2010). Curr. Opin. Drug Discovery Dev..

[cit170] Marwein S., Mishra B., De U. C., Acharya P. C. (2019). Curr. Pharm. Des..

[cit171] Gatta F., Del Giudice M. R., Borioni A., Borea P. A., Dionisotti S., Ongini E. (1993). Eur. J. Med. Chem..

[cit172] Jeong L. S., Jin D. Z., Kim H. O., Shin D. H., Moon H. R., Gunaga P., Chun M. W., Kim Y.-C., Melman N., Gao Z.-G. (2003). J. Med. Chem..

[cit173] Iannone R., Miele L., Maiolino P., Pinto A., Morello S. (2014). Am. J. Cancer Res..

[cit174] Beavis P. A., Divisekera U., Paget C., Chow M. T., John L. B., Devaud C., Dwyer K., Stagg J., Smyth M. J., Darcy P. K. (2013). Proc. Natl. Acad. Sci. U. S. A..

[cit175] Jin D., Fan J., Wang L., Thompson L. F., Liu A., Daniel B. J., Shin T., Curiel T. J., Zhang B. (2010). Cancer Res..

[cit176] Beavis P. A., Milenkovski N., Henderson M. A., John L. B., Allard B., Loi S., Kershaw M. H., Stagg J., Darcy P. K. (2015). Cancer Immunol. Res..

[cit177] Baraldi P. G., Manfredini S., Simoni D., Zappaterra L., Zocchi C., Dionisotti S., Ongini E. (1994). Bioorg. Med. Chem. Lett..

[cit178] Willingham S., Ho P., Leone R., Choy C., Powell J., McCaffery I., Miller R. (2016). Ann. Oncol..

[cit179] McCaffery I., Laport G., Hotson A., Willingham S., Patnaik A., Beeram M., Miller R. (2016). Ann. Oncol..

[cit180] Venugopala K. N. (2022). Pharmaceuticals.

[cit181] Churov A., Zhulai G. (2021). Hum. Immunol..

[cit182] Mediavilla-Varela M., Castro J., Chiappori A., Noyes D., Hernandez D. C., Allard B., Stagg J., Antonia S. J. (2017). Neoplasia.

[cit183] Merighi S., Battistello E., Giacomelli L., Varani K., Vincenzi F., Borea P. A., Gessi S. (2019). Expert Opin. Ther. Targets.

[cit184] de Goede K. E., Driessen A. J. M., Van den Bossche J. (2020). Biology.

[cit185] Deb P. K., Maity P., Sarkar B., Venugopala K. N., Tekade R. K., Batra S. ACS Pharmacol. Transl. Sci..

[cit186] Subudhi S., Falchook G. S., Salkeni M. A., El-Khoueiry A., Grewal J., Tester W., Pachynski R., Upadhaya S., Ibanez A. R. S., Kumar S. (2023). BMJ Specialist Journals.

[cit187] Chiappori A., Williams C., Creelan B., Tanvetyanon T., Gray J., Haura E., Chen D. T., Thapa R., Beg A., Boyle T. (2018). J. Thorac. Oncol..

[cit188] Harshman L. C., Chu M., George S., Hughes B. G. M., Carthon B. C., Fong L., Merchan J. R., Kwei L., Hotson A. N., Mobasher M., Miller R. A. (2020). J. Clin. Oncol..

[cit189] Powderly J., Spira A., Gutierrez R., DiRenzo D., Udyavar A., Karakunnel J. J., Rieger A., Colabella J., Lai D. W., de Souza P. (2019). Ann. Oncol..

[cit190] Zhang J., Yan W., Duan W., Wüthrich K., Cheng J. (2020). Pharmaceuticals.

[cit191] Lim E. A., Bendell J. C., Falchook G. S., Bauer T. M., Drake C. G., Choe J. H., George D. J., Karlix J. L., Ulahannan S., Sachsenmeier K. F. (2022). Clin. Cancer Res..

[cit192] Abo-Salem O. M., Hayallah A. M., Bilkei-Gorzo A., Filipek B., Zimmer A., Müller C. E. (2004). J. Pharmacol. Exp. Ther..

[cit193] Schneider G., Glaser T., Lameu C., Abdelbaset-Ismail A., Sellers Z. P., Moniuszko M., Ulrich H., Ratajczak M. Z. (2015). Mol. Cancer.

[cit194] Dungo R., Deeks E. D. (2013). Drugs.

[cit195] Deb P. K., Chandrasekaran B., Mailavaram R., Tekade R. K., Jaber A. M. Y. (2019). Drug Discovery Today.

[cit196] Zeng D., Maa T., Wang U., Feoktistov I., Biaggioni I., Belardinelli L. (2003). Drug Dev. Res..

[cit197] Jemal A., Siegel R., Xu J., Ward E. (2010). Ca-Cancer J. Clin..

[cit198] Mittal D., Sinha D., Barkauskas D., Young A., Kalimutho M., Stannard K., Caramia F., Haibe-Kains B., Stagg J., Khanna K. K. (2016). Cancer Res..

[cit199] Cheung A. W.-H., Brinkman J., Firooznia F., Flohr A., Grimsby J., Lou Gubler M., Guertin K., Hamid R., Marcopulos N., Norcross R. D. (2010). Bioorg. Med. Chem. Lett..

[cit200] Kaji W., Tanaka S., Tsukimoto M., Kojima S. (2014). J. Toxicol. Sci..

[cit201] Seitz L., Jin L., Leleti M., Ashok D., Jeffrey J., Rieger A., Tiessen R. G., Arold G., Tan J. B. L., Powers J. P. (2019). Invest. New Drugs.

[cit202] V Evans J., Suman S., Goruganthu M. U. L., Tchekneva E. E., Guan S., Arasada R. R., Antonucci A., Piao L., Ilgisonis I., Bobko A. A. (2023). JNCI, J. Natl. Cancer Inst..

[cit203] Jacobson K. A. , Klutz A. M., Tosh D. K., Ivanov A. A., Preti D. and Baraldi P. G., Medicinal Chemistry of the A3 Adenosine Receptor: Agonists, Antagonists, and Receptor Engineering, Adenosine Receptors in Health and Disease, 2009, pp. 123–159 10.1007/978-3-540-89615-9_5 PMC3413728 19639281

[cit204] Gallo-Rodriguez C., Ji X., Melman N., Siegman B. D., Sanders L. H., Orlina J., Fischer B., Pu Q., Olah M. E. (1994). J. Med. Chem..

[cit205] Kim H. O., Ji X., Siddiqi S. M., Olah M. E., Stiles G. L., Jacobson K. A. (1994). J. Med. Chem..

[cit206] Fishman P., Jacobson K. A., Ochaion A., Cohen S., Bar-Yehuda S. (2007). Immunol., Endocr. Metab. Agents Med. Chem..

[cit207] V Joshi B., Jacobson K. A. (2005). Curr. Top. Med. Chem..

[cit208] Bar-Yehuda S., Madi L., Silberman D., Gery S., Shkapenuk M., Fishman P. (2005). Neoplasia.

[cit209] Merighi S., Benini A., Mirandola P., Gessi S., Varani K., Leung E., Maclennan S., Borea P. A. (2005). J. Biol. Chem..

[cit210] Lu J., Pierron A., Ravid K. (2003). Cancer Res..

[cit211] Ohana G., Bar-Yehuda S., Arich A., Madi L., Dreznick Z., Rath-Wolfson L., Silberman D., Slosman G., Fishman P. (2003). Br. J. Cancer.

[cit212] Kim G. D., Oh J., Jeong L. S., Lee S. K. (2013). Biochem. Biophys. Res. Commun..

[cit213] Cohen S., Stemmer S. M., Zozulya G., Ochaion A., Patoka R., Barer F., Bar-Yehuda S., Rath-Wolfson L., Jacobson K. A., Fishman P. (2011). J. Cell. Physiol..

[cit214] Lee E.-J., Min H.-Y., Chung H.-J., Park E.-J., Shin D.-H., Jeong L. S., Lee S. K. (2005). Biochem. Pharmacol..

[cit215] Chung H., Jung J.-Y., Cho S.-D., Hong K.-A., Kim H.-J., Shin D.-H., Kim H., Kim H. O., Shin D. H., Lee H. W. (2006). Mol. Cancer Ther..

[cit216] Nakamura K., Yoshikawa N., Yamaguchi Y. U., Kagota S., Shinozuka K., Kunitomo M. (2006). Anticancer Res..

[cit217] Nakamura K., Shinozuka K., Yoshikawa N. (2015). J. Pharmacol. Sci..

[cit218] Blad C. C., von Frijtag Drabbe Künzel J. K., de Vries H., Mulder-Krieger T., Bar-Yehuda S., Fishman P., IJzerman A. P. (2011). Purinergic Signalling.

[cit219] Polycarpou E., Meira L. B., Carrington S., Tyrrell E., Modjtahedi H., Carew M. A. (2013). Mol. Nutr. Food Res..

[cit220] Aires V., Limagne E., Cotte A. K., Latruffe N., Ghiringhelli F., Delmas D. (2013). Mol. Nutr. Food Res..

[cit221] Ayoub B. M., Attia Y. M., Ahmed M. S., Enzyme J. (2018). Inhib. Med. Chem..

[cit222] Gessi S., Merighi S., Varani K., Leung E., Mac Lennan S., Borea P. A. (2008). Pharmacol. Ther..

[cit223] Gao Z., Li B.-S., Day Y.-J., Linden J. (2001). Mol. Pharmacol..

[cit224] Kreckler L. M., Wan T. C., Ge Z.-D., Auchampach J. A. (2006). J. Pharmacol. Exp. Ther..

[cit225] Gao Z.-G., Suresh R. R., Jacobson K. A. (2021). Purinergic Signalling.

[cit226] Kim H., Kang J. W., Lee S., Choi W. J., Jeong L. S., Yang Y., Hong J. T., Do Young Y. (2010). Anticancer Res..

[cit227] Spinaci A., Buccioni M., Dal Ben D., Maggi F., Marucci G., Francucci B., Santoni G., Lambertucci C., Volpini R. (2022). Pharmaceuticals.

[cit228] Federico S., Persico M., Trevisan L., Biasinutto C., Bolcato G., Salmaso V., Da Ros T., Gianferrara T., Prencipe F., Kachler S. (2023). ChemMedChem.

[cit229] Jacobson K. A., Park K.-S., Jiang J.-L., Kim Y.-C., Olah M. E., Stiles G. L., Ji X.-D. (1997). Neuropharmacology.

[cit230] Kim G., Hou X., Byun W. S., Kim G., Jarhad D. B., Lee G., Hyun Y. E., Yu J., Lee C. S., Qu S. (2023). J. Med. Chem..

[cit231] Sun C., Wang B., Hao S. (2022). Front. Immunol..

[cit232] Willingham S. B., Ho P. Y., Hotson A., Hill C., Piccione E. C., Hsieh J., Liu L., Buggy J. J., McCaffery I., Miller R. A. (2018). Cancer Immunol. Res..

[cit233] Hodgson R. A., Bertorelli R., Varty G. B., Lachowicz J. E., Forlani A., Fredduzzi S., Cohen-Williams M. E., Higgins G. A., Impagnatiello F., Nicolussi E. (2009). J. Pharmacol. Exp. Ther..

[cit234] Borodovsky A., Wang Y., Ye M., Shaw J. C., Sachsenmeier K. F., Deng N., DelSignore K. J., Fretland A. J., Clarke J. D., Goodwin R. J. (2017). Cancer Res..

[cit235] Buisseret L., Rottey S., De Bono J. S., Migeotte A., Delafontaine B., Manickavasagar T., Martinoli C., Wald N., Rossetti M., Gangolli E. A., Wiegert E. (2021). J. Clin. Oncol..

[cit236] Pastore D. R. E., Mookhtiar K., Schwartz B., Kumar S., Nagaraj R., V Meru A. (2022). Cancer Res..

[cit237] Xia C., Yin S., To K. K. W., Fu L. (2023). Mol. Cancer.

[cit238] Sachsenmeier K. , Sult E., Hay C. and Poon E., Therapeutic combinations comprising anti-cd73 antibodies and uses thereof, US Pat., US14/937565, MedImmune Ltd, 2016

